# Applicability of Recombinant Laccases From the White-Rot Fungus *Obba rivulosa* for Mediator-Promoted Oxidation of Biorefinery Lignin at Low pH

**DOI:** 10.3389/fbioe.2020.604497

**Published:** 2020-12-17

**Authors:** Jussi Kontro, Riku Maltari, Joona Mikkilä, Mika Kähkönen, Miia R. Mäkelä, Kristiina Hildén, Paula Nousiainen, Jussi Sipilä

**Affiliations:** ^1^Department of Chemistry, Faculty of Science, Chemicum, University of Helsinki, Helsinki, Finland; ^2^Department of Microbiology, Faculty of Agriculture and Forestry, Viikki Biocenter 1, University of Helsinki, Helsinki, Finland

**Keywords:** biorefinery lignin, oxidation, laccase, mediator, acidic conditions, structural analysis

## Abstract

Utilization of lignin-rich side streams has been a focus of intensive studies recently. Combining biocatalytic methods with chemical treatments is a promising approach for sustainable modification of lignocellulosic waste streams. Laccases are catalysts in lignin biodegradation with proven applicability in industrial scale. Laccases directly oxidize lignin phenolic components, and their functional range can be expanded using low-molecular-weight compounds as mediators to include non-phenolic lignin structures. In this work, we studied in detail recombinant laccases from the selectively lignin-degrading white-rot fungus *Obba rivulosa* for their properties and evaluated their potential as industrial biocatalysts for the modification of wood lignin and lignin-like compounds. We screened and optimized various laccase mediator systems (LMSs) using lignin model compounds and applied the optimized reaction conditions to biorefinery-sourced technical lignin. In the presence of both N–OH-type and phenolic mediators, the *O. rivulosa* laccases were shown to selectively oxidize lignin in acidic reaction conditions, where a cosolvent is needed to enhance lignin solubility. In comparison to catalytic iron(III)–(2,2,6,6-tetramethylpiperidin-1-yl)oxyl (TEMPO) oxidation systems, the syringyl-type lignin units were preferred in mediated biocatalytic oxidation systems.

## Introduction

Environmental concerns such as increasing fossil carbon footprint set demands for the development of more sustainable global energy and chemical flows. Lignocellulosic biomass, or plant dry matter, is a valuable renewable source for production of fuels, chemicals, and energy. Its main components are cellulose (38–50%), hemicelluloses (23–32%), and lignin (12–25%) (Ponnusamy et al., 2019). Lignin is an amorphous polymer that occurs in the cell walls of woody plants where it acts as a glue reinforcing structural integrity and provides a hydrophobic surface allowing water transport (Boerjan et al., 2003). Carbohydrate components of the plant cell walls consist of monomeric subunits linked through glycosidic bonds, making their polymeric structures more regular and relatively easy to cleave through either enzymatic or chemical catalysis. Lignin differs chemically from plant carbohydrates, being an aromatic polymer with monomeric units linked through a variety of ether and carbon–carbon linkages, such as aryl glycerol-β-aryl ether (β-O-4), phenylcoumaran (β-5), and resinol-type (β-β) bonds, to form a polymeric structure. Breaking these bonds requires, e.g., redox, pyrolysis, or hydrotreatment reactions (Boerjan et al., 2003; Xu et al., 2014).

Pulp and paper industries produce ~50 million tons of technical lignins each year (Bruijnincx et al., 2015), and estimations based on the investments and expansion of circular economy suggest that by the year 2050, lignocellulosic bioethanol plants will annually produce ~200 million tons of lignin as side product (IEA, 2009). Currently, the potential of lignin is underutilized in the industry, as most of it is burned for energy (Cao et al., 2018). Valorization of these lignin side streams potentially brings new business opportunities for second-generation biorefineries and supports their profitability. Lignin has potential for production of low-cost carbon fibers, engineering plastics, polymeric foams and membranes, and fuels and chemicals, all of which currently originate from nonrenewable resources (Zakzeski et al., 2010). Lignin-sourced products must be inexpensive, and their performance should be comparable to their petroleum-based counterparts before becoming economically and environmentally viable options. One of the suggested strategies in achieving this involves enzymatic biocatalysis, for which especially laccases show high potential (Mate and Alcalde, 2017).

Laccases (EC 1.10.3.2, *p*-diphenol:dioxygen oxidoreductases) are ubiquitous multi-copper enzymes found in plants, fungi, insects, archaea, and bacteria. Fungi, especially basidiomycetes, are the most prolific laccase producers in nature. Laccases mainly oxidize phenolic compounds through a relatively nonselective mechanism that involves four copper atoms at their active site (Baldrian, 2006). They do not utilize any organic cofactors or peroxides in their catalytic cycle; only molecular oxygen is required followed by production of water as a side product. This makes laccases very appealing biocatalysts for industrial processes (Virk et al., 2012). Heterologous expression of laccases in optimized hosts with possibility to genetically tailor their properties has been an important breakthrough for their industrial use, e.g., in bioremediation of soil and water xenobiotics, pulping and bleaching of lignocellulosic biomass, biosensors, and organic synthesis (Piscitelli et al., 2010).

Laccases have relatively low oxidation potentials (≤0.8 V) that limit their use to phenolic lignin substructures which comprise <20% of native lignin (Cañas and Camarero, 2010). Fungal laccases usually have higher redox potentials (+0.8 V) than plant or bacterial laccases (Baldrian, 2006). It has been shown that the oxidation of non-phenolic or bulky substrates is not possible by laccase alone, but the substrate range of laccases can be expanded indirectly through the use of small-molecular-weight mediator compounds that enable new reaction mechanisms for the selective oxidation of non-phenolic lignin benzylic side-chain moieties (Morozova et al., 2007; Cañas and Camarero, 2010). The most common N–OH-type mediators and phenolic mediators use a hydrogen atom transfer (HAT) mechanism with neutral radical species in their transition state (Morozova et al., 2007). The common chemical oxidant (2,2,6,6-tetramethylpiperidin-1-yl)oxyl (TEMPO), which is also a laccase substrate, oxidizes by an ionic mechanism, and the steric effects restrict the outcome of the benzylic oxidation reaction (Shiraishi et al., 2013). 2,2′-Azino-bis(3-ethylbenzothiazoline-6-sulfonic acid) (ABTS), which was the first mediator tested for kraft lignin (Bourbonnais and Paice, 1990), uses an electron transfer mechanism and has also been reported to cleave the Cα-Cβ bond in β-O-4-structures, which are the most abundant lignin structures (Morozova et al., 2007).

Laccases and laccase mediator systems (LMSs) have proven effects in pulp delignification (Camarero et al., 2007), pulp grafting (Aracri et al., 2010), and pitch removal (del Río et al., 2004; Gutiérrez et al., 2006). The LMSs have been applied on different types of lignins, e.g., alkali lignin, soda lignin, kraft lignin, organosolv lignin, and lignosulfonate, that all have different properties that affect the reaction outcome (Huber et al., 2016; Gasser et al., 2017; Xie et al., 2017; Longe et al., 2018). Depending on the lignin type, they can depolymerize, polymerize, and oxidize under LMS conditions. Primarily, LMS has been used to selectively oxidize the benzylic α-position of the non-phenolic lignin β-ether structures yielding the corresponding α-carbonyl structures that are chemically more reactive. The oxidized lignin structures have been targeted for depolymerization to produce value-added small molecules, e.g., by formic acid (Rahimi et al., 2014), zinc mediation (Lancefield et al., 2015), gold nanoparticles (Song et al., 2019), alkaline hydrogen peroxide (Gierer et al., 1977), or enzymatic catalysis (Mnich et al., 2017; Picart et al., 2017; Marinović et al., 2018). However, more work is needed to develop stable and tolerant laccases with reduced production costs in order to introduce effective oxidative biocatalytic processes for lignin valorization in the extreme reaction conditions required in cellulosic biorefineries. Also, development of improved mediators with increased stability is required for industrial process conditions (Virk et al., 2012).

Fungal laccases generally have an optimum pH around 4 for phenolic compounds (Baldrian, 2006). *Obba rivulosa* (syn. *Physisporinus rivulosus* and *Ceriporiopsis rivulosa*) is a basidiomycetous white-rot fungus that efficiently and selectively degrades softwood lignin in nature (Hakala et al., 2004). In addition to lignin-degrading peroxidases, it produces several laccase isoenzymes as a part of its lignin-degrading system (Miettinen et al., 2016). Two of these isoenzymes, *Or*Lcc1 and *Or*Lcc2, have demonstrated interesting properties, such as an extremely low optimum pH for phenolic compounds (<3.5 for 2,6-dimethoxy phenol) as well as improved performance in moderately elevated temperatures, that may prove beneficial in biorefinery applications (Hildén et al., 2007, 2013). The protein architecture at the reducing substrate-binding pocket near the T1-Cu site of *Or*Lcc1 and *Or*Lcc2 indicates the presence of four conserved amino acid substitutions in the structural models that contribute to the organic substrate-binding cavity. Both *Or*Lcc1 and *Or*Lcc2 are unable to oxidize the typical laccase substrate syringaldazine, which suggests a highly restricted T1-Cu site (Hildén et al., 2013). Also, the *O. rivulosa* laccases have been found to oxidize nonselectively both guaiacyl (G)- and syringyl (S)-type phenols (Hildén et al., 2007).

In this work, we studied the performance of *Or*Lcc1 and *Or*Lcc2 with various laccase mediators for oxidation of biorefinery lignin in low-pH environments using organic cosolvents to improve lignin solubility. The preference and efficiency of different LMSs were studied first using lignin model compounds. Among the mediators, especially 1-hydroxybenzotriazole (HBT), violuric acid (VIO), and syringyl nitrile (SCN) performed the best for selective benzylic oxidation to obtain corresponding carbonyls. The LMS was then applied to pre-purified biorefinery-sourced poplar lignin fractions. The analysis results show that the LMS works even under acidic conditions to oxidize both lignin model compounds and biorefinery lignin, producing the desired α-oxidized structures for further lignin processing by chemical or biocatalytic reactions, e.g., to advanced biofuels, octane boosters, and platform chemicals.

## Materials and Methods

### Materials

**Figure 1:**
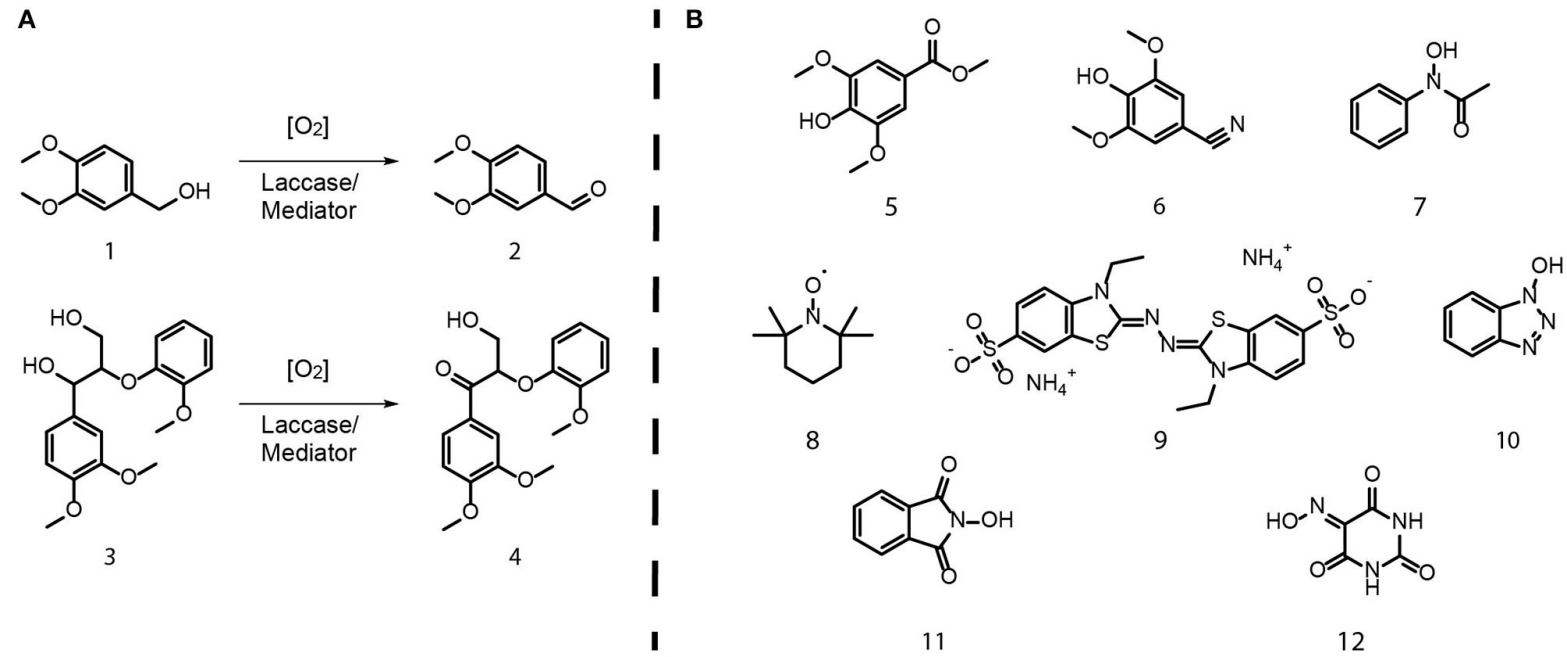
**(A)** The lignin model compounds **(1–4)** and the LMS oxidation experiments. **(B)** The mediator compounds **(5–12)** studied in LMS experiments.

Two laccases (*Or*Lcc1 and *Or*Lcc2) from white-rot fungus *O. rivulosa* were recombinantly produced in the methylotrophic yeast *Pichia pastoris* (Hildén et al., 2013). The structures of substrates and products are shown in Figure 1A and mediators in Figure 1B. The monomeric model compounds used, veratryl alcohol (**1**; Acros Organics) and veratraldehyde (**2**; Fluka), were of commercial grade. The dimeric arylglycerol β-ether compound 1-(3,4-dimethoxyphenyl)-2-(2-methoxy-phenoxy)-1,3-propanediol (**3**; as pure *erythro*) was synthesized following the procedure of Nakatsubo et al. (1975), and the corresponding ketone, 2-(2-methoxyphenoxy)-3-hydroxy-1-(3,4-dimethoxyphenyl)propan-1-one (**4**), was prepared following the methods of Adler et al. (1952) and Lahtinen et al. (2013). Methyl syringate (**5**; MeS) was prepared from syringic acid (Aldrich) by methylation in methanol using sulfuric acid as a catalyst. SCN (**6**, 3,5-dimethoxy-4-hydroxybenzonitrile) was synthesized from syringaldehyde in one-pot synthesis according to the method of Wang and Lin (1998), and *N*-hydroxyacetanilidine (**7**; NHA) was prepared following the procedure by Oxley et al. (1989). TEMPO (**8**), ABTS (**9**), 1-hydroxybenzotriazole (**10**; HBT), and *N*-hydroxyphthalimide (**11**; HPI) were purchased from Sigma-Aldrich, and VIO (**12**) was from Fluka. 1,3,5-Trimethoxybenzene (TMB), as an internal reference in high-performance liquid chromatography (HPLC), was from Fluka. Fe(NO_3_)_3_·9H_2_O was from Sigma-Aldrich. All the synthesized compounds were purified with silica column chromatography and characterized by nuclear magnetic resonance (NMR), before further use.

**Figure 2:**
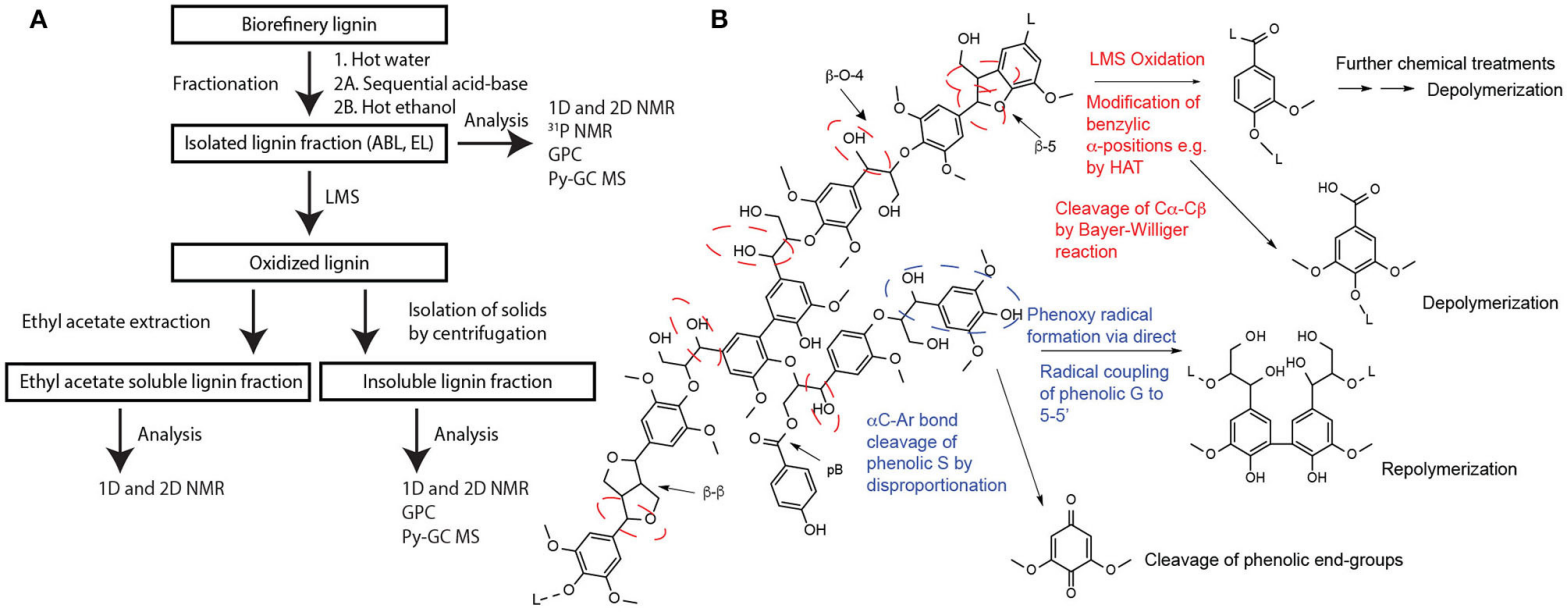
**(A)** The scheme for lignin sample treatments performed in this study and **(B)** the most prominent oxidative reactions based on literature (Du et al., 2013; Munk et al., 2015). Lignin tentative structure with its most abundant interconnecting linkages is depicted, and the prominent reactive sites in laccase-catalyzed redox and hydrogen transfer reactions are circled with dashed lines.

The lignin cake was received from Italian Bio Products SRL (Crescentino, Piedmont, Italy), where it was produced as a side product in the second-generation bioethanol process PROESA® technology with poplar as feedstock. According to the manufacturer's report, the raw lignin was characterized in terms of residual sugars (30 wt% on dry basis), Klason lignin (55 wt% on dry basis), ashes (2 wt% on dry basis), other (13 wt% on dry basis), and moisture content (67%). The lignin preparate was first heated at 80°C for 30 min in water to remove inorganic compounds and soluble carbohydrates and then filtered and dried. The washed lignin was then fractionated by two different methods prior to LMS experiments (Figure 2A). The first fractionation method, based on a method by Salanti et al. (2010), consisted of sequential treatments with mild acid (0.1 M HCl) and base (0.1 M NaOH) at 100°C for 2 h with filtration of the impurities and precipitation of purified lignin by acidification. This yielded 30% w/w carbohydrate-free lignin (acid–base purification ABL) with a lignin content of 90%, corresponding to 55% of the Klason lignin of the original lignin cake. In the second approach, lignin was fractionated by refluxing in absolute ethanol for 4 h followed by filtration of the hot solution with the Büchner funnel. The hot ethanol-soluble lignin (EL) fraction was 18% of the total mass with a lignin content of 95%, corresponding to 33% of the Klason lignin in the original lignin cake. Both ABL and EL were structurally characterized by NMR spectroscopy, infrared spectroscopy (IR), gel permeation chromatography (GPC), and pyrolysis gas chromatography–mass spectrometry (py-GC/MS) and qualitatively by elemental analysis and residual ash using thermogravimetric analysis (TGA).

The lignin contents of the fractions were analyzed using the acetyl bromide method (Hatfield et al., 1999; Hyväkkö et al., 2019), where lignin is derivatized and solubilized in acetic acid. UV absorptions of the samples were measured at 280 nm by using a Varian Cary 50 Conc UV–VIS spectrometer.

### Laccase Activity and Solvent Stability

The initial activity of *Or*Lcc1 and *Or*Lcc2 was determined by following the oxidation of phenolic substrate 0.1 mM 2,6-dimethoxy phenol (2,6-DMP) in 100 mM citrate-phosphate buffer at pH 3.0 to the corresponding dimeric product (λ = 468 nm, ε = 14,800 M^−1^ cm^−1^) at 25°C (Solano et al., 2001). Catalytic activity was expressed as nkat/ml for the formed product. The initial reaction rates and stabilities were also tested in varying solvent systems and pH. The effect of the cosolvent on the oxidation of 2,6-DMP was studied in 1,4-dioxane (20% v/v) and ethanol (20%, 40%, and 50% v/v) at pH 3.0, 4.0, and 5.0 by adding the enzyme preparate to the mixtures that already contained the solvent and substrate and immediately measuring the absorbance for 2 min. The initial laccase activities were calculated, and these values were used when the laccases were dosed into oxidation experiments with lignin model compounds and lignins.

Solvent stability of the laccases was deducted from residual activity toward 0.5 mM ABTS in sodium malonate buffer (pH 3.0) after 2.5-h incubation in *n*-butanol (15–20%), 1,4-dioxane (20–40%), and ethanol (10–40%).

### Laccase-Catalyzed Oxidation of Lignin Model Compounds in the Presence of Mediators

The oxidation mixtures with veratryl alcohol (**1**) or 2-(2-methoxyphenoxy)-3-(3,4-dimethoxyphenyl)propan-1,3-diol **(3)** (Figure 1) were gently shaken in an Eppendorf Thermomixer C 500 rpm at 25°C in 15-ml screw-cap tubes to ensure sufficient oxygen. Each reaction mixture (0.5 or 1 ml) contained 6 or 12 mM of substrate (**1** or **3**); 6 or 12 mM of mediator (**5**–**12**), with or without internal standard TMB (12 mM); and 10 or 20 nkat/ml of laccase (*Or*Lcc1 or *Or*Lcc2). In order to get comparable results with *Or*Lcc1 and *Or*Lcc2, the substrate-to-mediator ratio of 1:1 was used in all experiments, and also the laccase activity-to-substrate ratio was kept constant (20 nkat/ml vs. 12 mM substrate or 10 nkat/ml vs. 6 mM substrate). The solvent system consisted of a mixture of 100 mM citrate-phosphate buffer pH 3.0 and contained 20% 1,4-dioxane or 40% ethanol (v/v). Samples for HPLC analysis were taken at 0, 2, 4, 6, 24, and 48 h.

### Analysis of the Reaction Mixtures by HPLC

The oxidation of veratryl alcohol (**1**) and 2-(2-methoxyphenoxy)-3-(4-hydroxy-3-methoxyphenyl)propan-1,3-diol (**3**) was monitored by HPLC Agilent 1200 (Santa Clara, CA, USA) as described earlier (Nousiainen et al., 2014). The samples were prepared by taking a 50-μl sample and diluting it with 1 mg/ml NaN_3_ in a methanol:water (60:40) mixture to inhibit further reactions. A sample of 5 μl was injected with an autosampler and eluted with a MeOH:H_2_O (Milli-Q) gradient with a 0.25-ml/min flow rate: 0–1 min 38:62 isocratic conditions; 1–7 min linear gradient from 38:62 to 100:0; and 7–18 min 100:0 to wash the column. The column was stabilized with 18–21 min linear gradient from 100:0 to 38:62 and in 21–31 min 38:62 isocratic conditions.

### Oxidation of Biorefinery Lignin

The experiments with biorefinery lignin were performed on previously described ABL and EL lignin preparates (see Section materials and Supplementary Table 1). Typically, 200-mg lignin samples were weighed into 15-ml plastic centrifuge tubes and suspended into 10 ml of solvent (20% 1,4-dioxane in citrate-phosphate buffer, pH 3.5). Then, 0.15 mmol of mediator HBT (**10**; 20 mg), SCN (**6**; 27 mg), VIO (**12**; 26 mg), or TEMPO (**8**; 23 mg) was added. Control samples with and without laccase were also included. Reactions were initiated by adding 50 μl of *Or*Lcc2 (35 nkat based on initial 2,6-DMP activity at pH 3.5). The tubes were plugged using septa pierced with needles and slowly flushed with air to keep the solution aerated during the reaction. The reactions were shaken in a Thermomixer C 600 rpm for 72 h at 25°C. Liquid samples were concentrated to half of the initial volume and acidified to pH 1.5 by adding 1 M HCl with subsequent formation of solid precipitates. All samples were centrifuged (3,200 × *g*, 10 min, 20°C) to collect the precipitates. Pellets were washed twice with 10 ml water to remove salts and buffers and further extracted with 2 × 5 ml ethyl acetate to remove mediators. Finally, the solid materials were dried under argon flow and prepared for analysis, and they were referred to as insoluble lignin fractions as shown in Figure 2A. These samples were analyzed by NMR, GPC, IR, and py-GC/MS. The ethyl acetate-soluble fractions were combined and used to extract the aqueous supernatants and then evaporated to dryness and were referred to as organic solvent-soluble lignin fractions in Figure 2A. These samples were analyzed by NMR.

For NMR and GPC analyses, the solid samples were acetylated by incubating in a 1:1 mixture of acetic anhydride and pyridine overnight at 50°C. The excess reagents were quenched with ethanol, and the solvents were removed by azeotropic distillation with toluene under reduced pressure using a rotary evaporator.

In order to study the effect of excess oxygen, four 300-mg lignin samples were suspended in 2 ml of 1,4-dioxane and mixed under magnetic stirring. Either 0.12 mmol of SCN (**6**) or 0.12 mmol of VIO (**12**) was added to the mixtures. All samples were diluted with 8 ml of citrate-phosphate buffer, pH 3.0, mixed, and finally, *Or*Lcc2 (100 μl, 110 nkat) was added into all except the control sample. The reaction flasks were purged in vacuum and then flushed with oxygen three times. This procedure was repeated once an hour for 5 h. The reaction mixtures were stirred under oxygen atmosphere for 24 h and then concentrated to remove 1,4-dioxane. The remaining mixtures were transferred to centrifuge tubes with 2 M HCl and centrifuged to collect precipitates. The solids were washed with water until neutral. The combined supernatants were extracted with ethyl acetate, and the extracts were evaporated to dryness under reduced pressure. The solids from each reaction were combined with corresponding ethyl acetate fractions, and the total yields varied between 75 and 90%.

Modified TEMPO-based (Rahimi et al., 2013) catalytic oxidation procedures involving Fe(III)(NO_3_)_3_ either in 1,4-dioxane or in formic acid were applied to EL. Lignin (210 mg) was dissolved in 1,4-dioxane (11 ml), and TEMPO (**8**; 31 mg, 0.2 mmol), Fe(NO_3_)_3_·9H_2_O (52 mg, 0.13 mmol), and NaCl (31 mg, 0.5 mmol) were added. The reaction mixture was stirred at room temperature under oxygen atmosphere for 72 h while purging with oxygen twice a day. After completion, reaction mixture was diluted with acetone and filtered through a small layer of silica to remove the salts. The product was isolated by evaporation with a 150-mg (71%) yield. In the second experiment, lignin (190 mg) was dissolved in formic acid (10 ml), and TEMPO (**8**; 28 mg, 0.2 mmol), Fe(NO_3_)_3_·9H_2_O (60 mg, 0.15 mmol), and NaCl (58 mg, 1 mmol) were added. The reaction mixture was stirred at room temperature while purged with air for 4 h and then left stirring for 24 h. After completion, water was added to precipitate lignin, which was separated by centrifugation and washed until neutral. The aqueous phase was extracted twice to ethyl acetate, and the extracts were evaporated to dryness. The pellet (120 mg, 63%) and extract (50 mg, 26%) were dried under vacuum and analyzed separately.

### GPC Analysis of Lignin Samples

Acetylated samples (1–4 mg) were dissolved into THF to a concentration of 1 mg/ml. The solutions were mixed under magnetic stirring for 20 h and then filtered through 0.2-μm GHP syringe filters (Waters). The GPC measurements were performed on a Waters Acquity APC (Advanced Polymer Chromatography) equipment using a set of Acquity APC™ XT 45 Å (1.7 μm, 4.6 × 150 mm) and XT 200 Å (2.5 μm, 4.6 × 150 mm) columns (Waters Corporation, Milford, USA) in a series. The analyses were run in THF with detection by UV (254 and 280 nm) and refractive index (RI). Data processing was performed using GPC Empower® software (Waters). Using a set of polystyrene standards (Scientific Polymer Products and Fluka Analytical), the relative molecular weights were calculated by the software, giving M_N_ (number-average molecular weight), M_W_ (weight-average molecular weight), and polydispersity index (PDI; M_W_/M_N_).

### NMR Spectroscopy Analysis of Lignin Samples

The acetylated samples were dissolved in acetone-*d*_6_. A Bruker Avance III 500-MHz FT-NMR spectrometer with a Bruker 5-mm BBO probe was used for ^1^H, ^13^C, heteronuclear single-quantum coherence (HSQC), and heteronuclear multiple-bond correlation (HMBC) experiments. The Bruker standard pulse sequences hsqcetgp for HSQC and hmbcqplndqf for HMBC were used in acquisition with 11.8- and 210-ppm spectral widths in F2 (^1^H) and F1 (^13^C), respectively. The ^13^C spectra were performed with a 90° flip angle with power-gated decoupling of ^1^H and a relaxation delay of 1.5 s. The spectra were processed with the Bruker TopSpin 4.0.5 software. Solvent signals (2.05/29.84 ppm) were used as reference. The peaks were assigned based on literature (Gottlieb et al., 1997; Ralph and Landucci, 2010; Rahimi et al., 2013; Balakshin and Capanema, 2015). The integration limits for the ^1^H spectral signals were selected as 7.7–7.2 ppm for the oxidized aromatic region and 7.2–6.3 ppm for the regular aromatic region based on Rahimi et al. (2014).

### Infrared Spectral Analysis of Lignin Samples

The IR spectra were measured from the non-acetylated samples using a Bruker Alpha FTIR spectrometer equipped with an ATR module for sampling. Small samples of solid products were placed on the measuring plate, and the spectra were measured in transmittance mode. Each measurement consisted of 16 scans with background correction. From each experiment, three samples were measured. The spectra were background corrected using asymmetric least squares fitting and then standardized using the Origin 2018 software.

### py-GC/MS Analysis of Lignin

The analytical-scale pyrolysis equipment Pyrolab2000 (Pyrol AB, Sweden) was adopted using a platinum foil pulse pyrolyzer and 580°C isothermal pyrolysis temperature under He atmosphere (Ohra-aho et al., 2005; Kuuskeri et al., 2016). The system was directly connected to a Bruker Scion SQ 456-GC/MS equipped with an Agilent DB-5MS UI (5%-phenyl)-methylpolysiloxane (30 m × 0.250 mm × 0.25 μm film) capillary column. The injector temperature was 250°C, ion source was 250°C with electron ionization of 70 eV, the MS scan range was *m*/*z* 40–400, and helium was the carrier gas at the flow rate of 1 ml/min and 1:2 split ratio. Products were identified with comparison to selected reference compounds with their retention times and mass spectra and by referencing the National Institute of Standards and Technology (NIST) spectral library and literature (Kuuskeri et al., 2016). AMDIS software (version 2.70, NIST, USA) was used for identification and deconvolution of peaks.

## Results and Discussion

### Activity and Stability of *O. rivulosa* Laccases in Solvent Systems

The recombinant *O. rivulosa* laccases, *Or*Lcc1, and *Or*Lcc2, shown to be stable under increased acidity and high process temperatures, suggesting their potential for biocatalytic applications in acidic reaction media such as in conversions of lignin, dyes, or other xenobiotics (Hildén et al., 2013), were selected as biocatalysts in this study.

The solubility of lignin-related materials and even low-molecular-weight model compounds is in most cases low in acidic aqueous solutions. Cosolvents, such as various alcohols, 1,4-dioxane, pyridine, DMSO, or ionic liquids, are typically used to increase the solubility of these materials and compounds and to facilitate catalytic reactions while maintaining mild reaction conditions (Schuerch, 1952; Schutyser et al., 2018; Dastpak et al., 2020). Therefore, the stability of the *O. rivulosa* laccases was first determined in the presence of organic cosolvent ethanol, 1-butanol, or 1,4-dioxane for 2.5 h. After the enzymes were pooled out from the solvent mixtures and the residual activities were measured, both laccases were found to tolerate the solvent mixtures well, as they retained their activity or were activated in the tested conditions (Supplementary Figure 1). In particular, the *Or*Lcc2 was strongly activated after 2.5-h incubation in 40% ethanol, and this is a known phenomenon after preincubation in organic solvents (Wu et al., 2019). The only exception was *Or*Lcc2, which lost half of its activity in 40% 1,4-dioxane.

**Figure 3:**
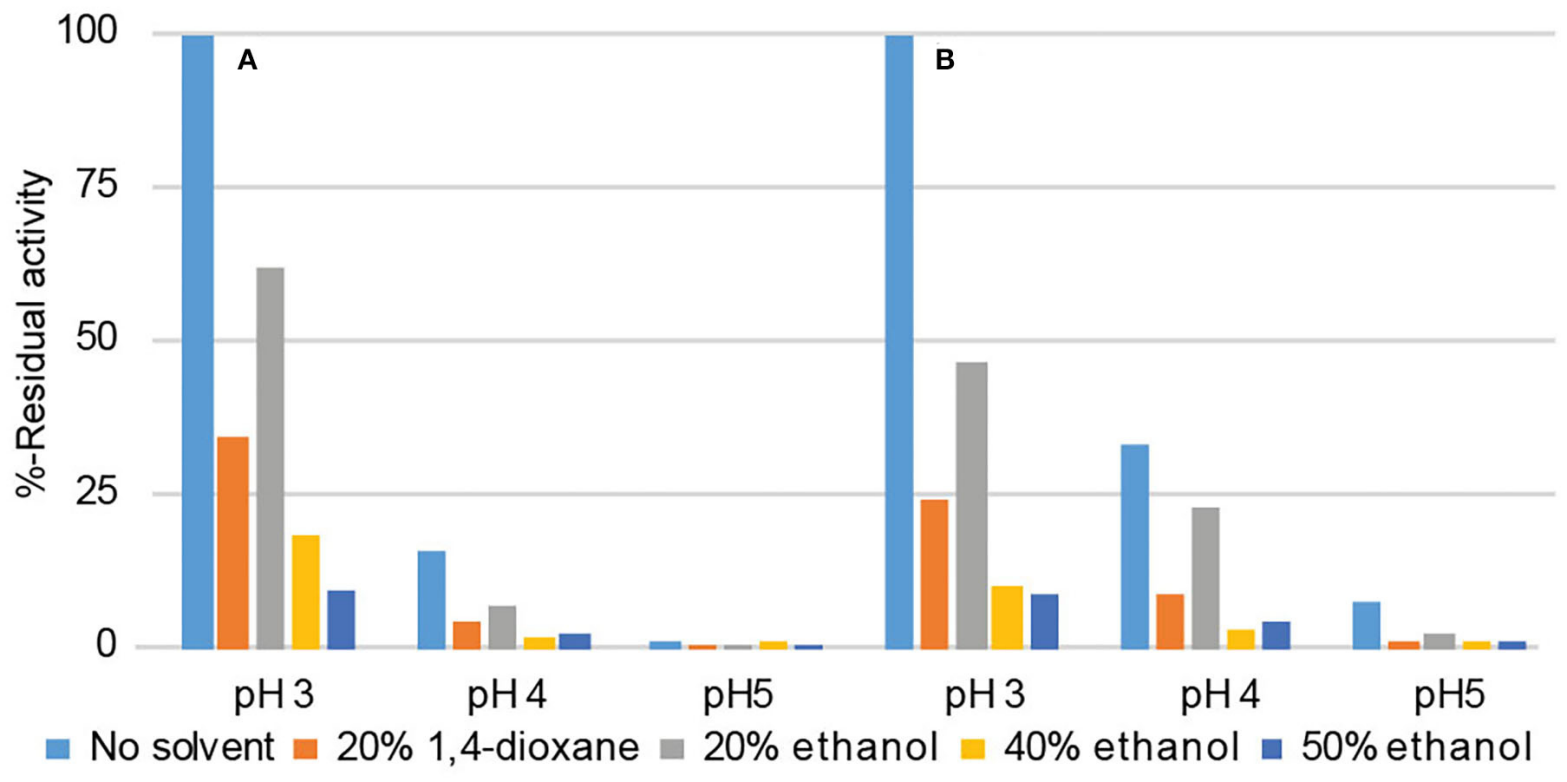
The effect of the solvent on the initial reaction rate of **(A)** *Or*Lcc1 and **(B)** *Or*Lcc2 with 2,6-DMP.

Different solvent systems affected the catalytic activity of both laccases when their initial activities were determined. The results are expressed as residual activities defined as a percentage of the initial activities of laccases assayed in aqueous buffer without organic solvents. In spite of the observed activation after preincubation conditions, all tested solvent systems were found to decrease the laccase activity, but the tolerance of the laccases for solvent systems varied (Figure 3). The optimal reaction conditions of laccase activity with respect to solubility of the substrates and residual activity of laccases were 1,4-dioxane (20%) at pH 3. In those conditions, *Or*Lcc1 and *Or*Lcc2 retained 34% and 24% of their activity, respectively. Although in ethanol (20%), the activities were notably higher than in 1,4-dioxane (62% for *Or*Lcc1 and 46% for *Or*Lcc2), ethanol concentration was not high enough to keep the model compounds in solution during the LMS reactions. However, in higher ethanol concentration (40%), only 18% of the initial activity was achieved with *Or*Lcc1 and 10% with *Or*Lcc2.

### Oxidation of Model Compounds

Different types of mediators that exhibit different reaction mechanisms on their oxidation (Morozova et al., 2007) were tested to find out whether the acidic reaction conditions affect stabilities of the mediators. Veratryl alcohol (**1**) was used to screen the oxidative capacity of *O. rivulosa* laccases with a set of common (HBT, TEMPO, ABTS, HPI, VIO, and NHA) and target-synthesized phenolic mediators (MeS and SCN). This assay gives information on the laccase activity on the mediator as substrate and the stability of the LMS system. In order to examine the effect of the full β-O-4 side chain on benzylic oxidation, the LMS was then studied using the dimeric non-phenolic aryl glycerol β-ether model compound (**3**). Experiments with this compound mimic the oxidation of secondary benzylic alcohols in lignin to the corresponding ketone (**4**).

**Figure 4:**
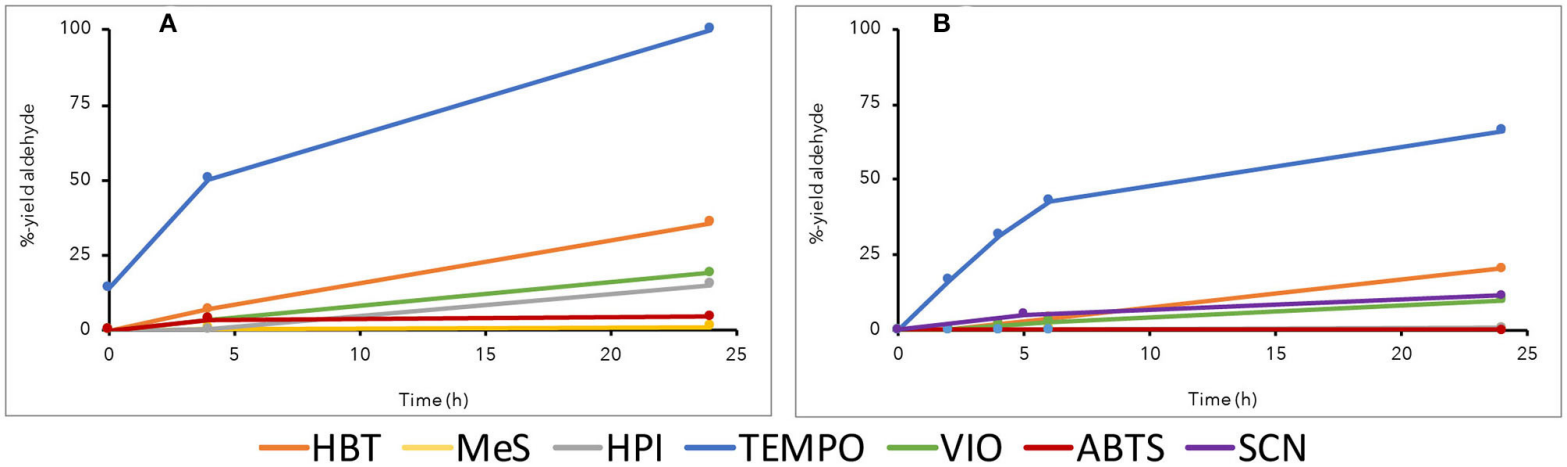
Oxidation of monomeric lignin model compound veratryl alcohol **(1)** to veratraldehyde **(2)** in various laccase mediator systems in 20% 1,4-dioxane at pH 3. **(A)** *Or*Lcc1 and **(B)** *Or*Lcc2.

**Figure 5:**
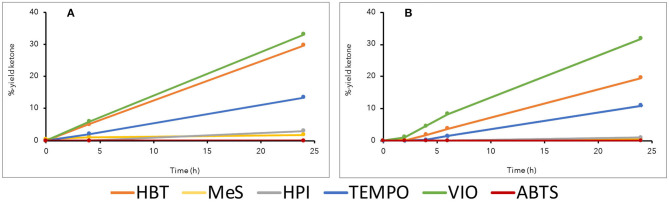
Oxidation of dimeric lignin β-O-4-aryl ether model compound **(3)** to the corresponding ketone **(4)** in various laccase mediator systems in 20% 1,4-dioxane at pH 3. **(A)** *Or*Lcc1 and **(B)** *Or*Lcc2.

With the model compound veratryl alcohol, *Or*Lcc1 showed slightly more efficient oxidation than did *Or*Lcc2, and the mediator preference for both laccases was TEMPO>HBT>VIO, SCN>HPI, MeS, and ABTS (Figure 4). The most effective oxidation of veratryl alcohol by both laccases was found when TEMPO was used as a mediator in 1,4-dioxane (20%): *Or*Lcc1 oxidized 100% and *Or*Lcc2 70% of veratryl alcohol to veratraldehyde (Figure 4). However, with the dimeric β-O-4-aryl ether model compound, the steric restrictions altered the mediator preferences. In particular, with TEMPO–LMS, the yields of the corresponding oxidized ketone product were much lower (13% for *Or*Lcc1 and 11% for *Or*Lcc2) (Figure 5), but a slight formation of veratraldehyde was detected, suggesting some ether bond cleavage through retro-aldol reaction (Sedai and Baker, 2014). Overall, in veratryl alcohol oxidation, HBT performed better than VIO, but with the β-O-4-dimer, VIO resulted in the highest ketone yields (32–34%) with both laccases, followed by HBT, TEMPO, and SCN. This suggested that VIO was a promising mediator with respect to oxidation of polymeric lignins.

The LMS oxidation involves electron transfer from the substrate to the laccase active site. Generally, in the electrochemical point of view, the basidiomycete laccases with high redox potential are able to oxidize substrates (and mediators) with higher oxidation potential (E^0^) more efficiently, resulting in higher oxidation rates. Low-redox-potential laccases are either unable to oxidize some N–OH-type mediators, like HBT [E^0^ 1.08 vs. normal hydrogen electrode (NHE)] and HPI (E^0^ 1.09 vs. NHE) (Barreca et al., 2004), or able to oxidize them very slowly. Laccases generally have higher formal oxidation potentials in acidic conditions (Xu, 1997), which may facilitate the oxidation of mediators, because a large oxidation potential gap between laccase and its substrate makes the oxidation reaction thermodynamically more demanding, resulting in slower reaction rates (Rochefort et al., 2004; Rodgers et al., 2010). The ability of the *O. rivulosa* laccases to readily oxidize mediators with high oxidation potentials suggests the high oxidation capacity of these enzymes.

The optimum pH for both laccases for 2,6-DMP was in the range of pH 3–4. In the mediated reactions, both the mediator stability and the enzyme's substrate specificity are also pH dependent and need to be taken into account in the reaction conditions. The mediated oxidation reactions were further investigated with *Or*Lcc2 using HBT and NHA at pH 3 and 4 and with phenolic mediators SCN and MeS at pH 4 and 5. We found that in most cases, the optimal reaction conditions were at pH 4 (Table 1), which is better for the stability of the phenolic mediator (Nousiainen et al., 2012) but suboptimal for *Or*Lcc2 (Figure 3). Phenolic mediators, like MeS, that usually perform best near neutral reaction conditions (Nousiainen et al., 2012) were found to be unstable in acidic conditions where acid-catalyzed side reactions consume the mediator, resulting in poor conversion. Even though MeS has been shown to be an effective mediator at neutral pH using both high and low-redox-potential laccases (Rosado et al., 2012; Rico et al., 2014), MeS resulted in low veratraldehyde yields after 24 h (<2%). However, the modified SCN proved to be relatively stable at pH 3–5 with *Or*Lcc2 producing 10–22% yield of the oxidized product (Table 1). SCN produced unwanted side products but to a lesser extent than other phenolic mediators (Nousiainen et al., 2012). When the reactions were followed for 120 h, up to 50% aldehyde was formed showing that both the laccase and mediator had high stability in these reaction conditions. The ABTS was found not to oxidize either of the model compounds under our experimental conditions, and specifically, no Cα-Cβ bond cleavage of the β-O-4-dimer was observed. However, in experiments that contained TMB (E^0′^ 1.5 V, Zweig et al., 1964) as internal standard, ABTS was found to oxidize the electron-rich TMB effectively (Supplementary Figure 2). With other mediators, oxidation of TMB was negligible.

**Table 1:** Oxidation of veratryl alcohol **(1)** and β-0-4-aryl ether dimer **(3)** by *Or*Lcc2 in 20% 1,4-dioxane at different pHs followed for 24 and 120 h.

		**Oxidation yield (%) against reaction time**			
		**veratraldehyde (2)**		**α-oxidized ketone dimer (4)**	
Mediator	pH	24 h	120 h	24 h	120 h
HBT	3	22	85	16	58
	4	22	100	19	61
NHA	3	21	58	11	26
	4	34	91	18	43
	5	14	68	n.d.	n.d.
SCN	3	10	17	n.d.	n.d.
	4	22	43	4	7
	5	11	50	1	9
MeS	4	8	10	n.d.	n.d.

*n.d. not determined*.

Both enzymes were also tested in buffered ethanol, but because ethanol has limited solvent capacity for both lignin and lignin model compounds, a high ethanol concentration (40%) was required. In particular, the activity of *Or*Lcc1 decreased considerably under these conditions, and only 1% veratraldehyde was obtained by *Or*Lcc1 with MeS and HBT and 2.5% with TEMPO in ethanol (40%) in 24 h. All the model compound reactions catalyzed by *Or*Lcc1 almost completely stopped already after 4 h, and practically no reaction occurred with β-O-4-dimer. Ethanol (40%) decreased the reaction rates of *Or*Lcc2, but they remained significantly higher compared to those of *Or*Lcc1, and up to 15% of veratraldehyde was formed with TEMPO in 24 h. In addition, the reactions proceeded steadily even after 24 h, showing the high solvent tolerance of this enzyme.

### Lignin Oxidation by Recombinant Laccases From *O. rivulosa*

The potential of *O. rivulosa* laccases for modifying biorefinery hardwood lignin was evaluated with and without mediators because both enzymes demonstrated good solvent tolerance and high LMS oxidation effect on the dimeric lignin model compound.

The material explored in the oxidation studies was a residual lignin-rich fraction obtained from the ethanolic biorefinery process isolated after steam explosion and saccharification phases of poplar wood. The raw material composition was heterogeneous, consisting of both G- and S-units and typical structural lignin units thereof. It also contained impurities such as a high amount of residual carbohydrates and possible condensation and polymerization side products referred to as pseudo-lignin (Shinde et al., 2018). This caused difficulties in analyzing the material and interpreting the results, and therefore, the raw material was fractionated before use in enzymatic reactions. The sequential acid–base purification produced lignin fraction (ABL) with an average M_W_ of 5.1 kDa (PDI 3.6), and it was used in the first set of LMS experiments. Different cosolvents (20% 1,4-dioxane and 40 and 50% ethanol) were tested to increase lignin solubility into reaction mixtures. Finally, a fraction obtained by extraction with hot ethanol (EL) with an average M_W_ of 2.2 kDa (PDI 2.0) was found to be a good starting material to serve as substrate in laccase oxidation experiments, even though it contained some wood extractives such as fatty acids.

Both laccases had a narrow working pH range revealed by initial activity measurements and model compound experiments that measure the mediated oxidation capacity. The reactions were made at both pH 3 and 4 to find out if either of the laccases had specific preferences for specific oxidation vs. polymerization of lignin depending on changes in pH reaction conditions (Supplementary Table 1). The fractionated biorefinery lignin was treated by *Or*Lcc2 with HBT, MeS, SCN, HPI, VIO, TEMPO, and ABTS at pH 3.0 and 4.0, which were close to the optimum pH of the enzyme. Accordingly, *Or*Lcc1 was tested with the most promising mediators, optimized for *Or*Lcc2, HBT, VIO, and SCN, at the optimum pH of 3.0. Analyses of the isolated insoluble LMS-oxidized lignins (Figure 2A) were divided into two parts: half of each sample was acetylated, and the other half was left untreated. From the non-acetylated samples, IR and py-GC/MS were analyzed. The molecular weight distributions (MWDs) were analyzed from the acetylated samples by GPC. Some of the acetylated samples were also analyzed by NMR to obtain further information on the structural changes in lignin molecule during the oxidation and to study the introduction of α-oxidized structures on lignin.

Because the laccase oxidation reaction requires oxygen to proceed, the low oxygen concentration may become a limiting factor to the progress of reaction (Huber et al., 2016). Mechanistically, it has been suggested that in addition to the laccase catalytic cycle, the mediated oxidation also consumes one equivalent of oxygen for each formed α-oxidized lignin phenylpropanoid unit (Crestini et al., 2003), increasing the oxygen demand further. Initially, the tubes were aerated by slow air flush, but the solvent levels were not possible to keep constant during the long reaction times. Thus, the oxidations were repeated in higher-volume reaction flasks under oxygen atmosphere, and in comparison to the earlier experiments, no major changes in the oxidation degree were observed, but a slight increase in molecular weight was found (Supplementary Figure 3).

Chemical oxidation reactions of lignin model compounds in highly acidic conditions have been shown to proceed in high yields according to Rahimi et al. (2013). For comparison, the chemical catalytic oxidation was applied to EL fractions by applying the method with high reported yields of secondary alcohol oxidation, the metal-catalyzed aerobic Fe(III)NO_3_-TEMPO system. Two efficient solvent systems, 1,4-dioxane and formic acid, that have shown good solvent properties for lignin were applied.

### GPC Elucidates the Molecular Weight Changes in Enzymatically Modified Lignins

**Figure 6:**
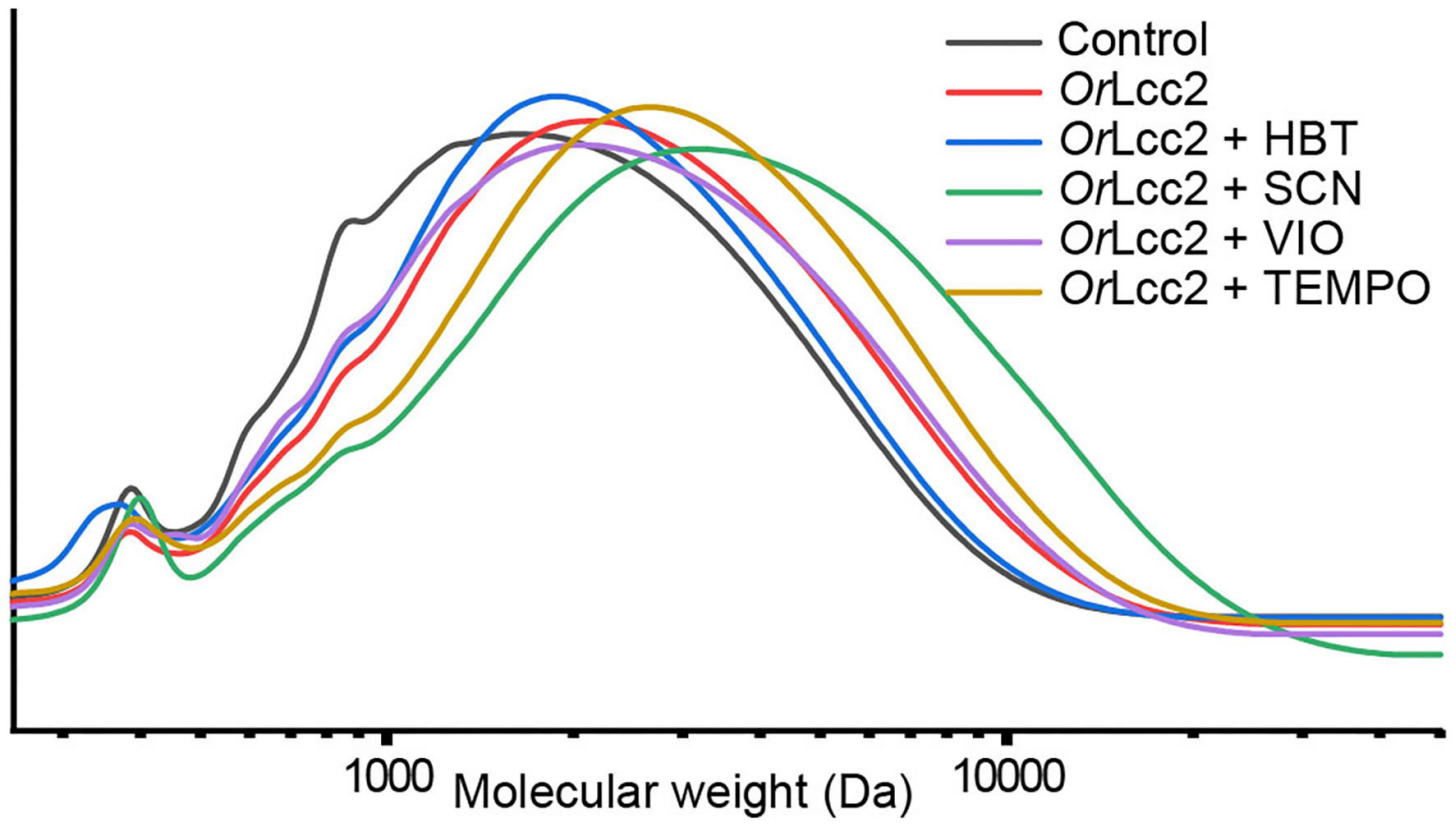
Effect of *Or*Lcc2 with different LMS treatments on the MWD of the hot ethanol-soluble lignin fraction in buffered 1,4-dioxane.

The laccase–mediator treatment did not impact the MWD of the samples to a large extent (Figure 6) as similarly suggested by earlier studies on selective lignin benzylic oxidation by 2,3-dichloro-5,6-dicyano-1,4-benzoquinone (DDQ) (Song et al., 2019).

The initial experiments in acidic pH 3 and 4 in buffered 1,4-dioxane (20%) and ethanol (50%) revealed only small changes in molecular weight in all analyses (data in Supplementary Table 1). The results varied from slight polymerization with ΔM_W_ 1–16% to some depolymerization with ΔM_W_ 8–25%. These small variations were probably due to changes in the chemical nature of the polymer substructures rather than condensation, or repolymerization or depolymerization. The oxidized mediators mainly transfer the oxidation to the benzylic lignin positions with α-oxidation of β-O-4-aryl ether substructures through the HAT mechanism (Morozova et al., 2007; Cañas and Camarero, 2010), although some direct laccase activity on lignin through single-electron oxidation has to be considered, such as polymerization (Munk et al., 2015), grafting of the mediator onto lignin (Munk et al., 2017), and breakdown of other (e.g., phenolic) lignin substructures (D'Acunzo et al., 2006) (Figure 2B). In LMS, depolymerization has been suggested to occur via the Bayer–Williger reaction (Du et al., 2013) with concomitant formation of carboxylic acid functionalities in lignin. Also, changes may be expected because in the isolation procedure, the mediator was extracted from the matrix with ethyl acetate, and according to ^1^H NMR analyses (Supplementary Figure 4), some low-molecular-weight lignin fragments were transferred to this fraction, affecting the results. In general, with the ABL lignin fraction, the lower-pH (pH 3) oxidation systems were found to be more effective, and the structural analyses (py-GC/MS and IR) showed promising signs of α-oxidation in the material. However, the low solubility of ABL in the 20% aqueous dioxane was suspected as an obstacle for laccase activity on lignin, and to improve solubility of lignin, a higher amount of ethanol (50%) was used as a cosolvent.

**Figure 7:**
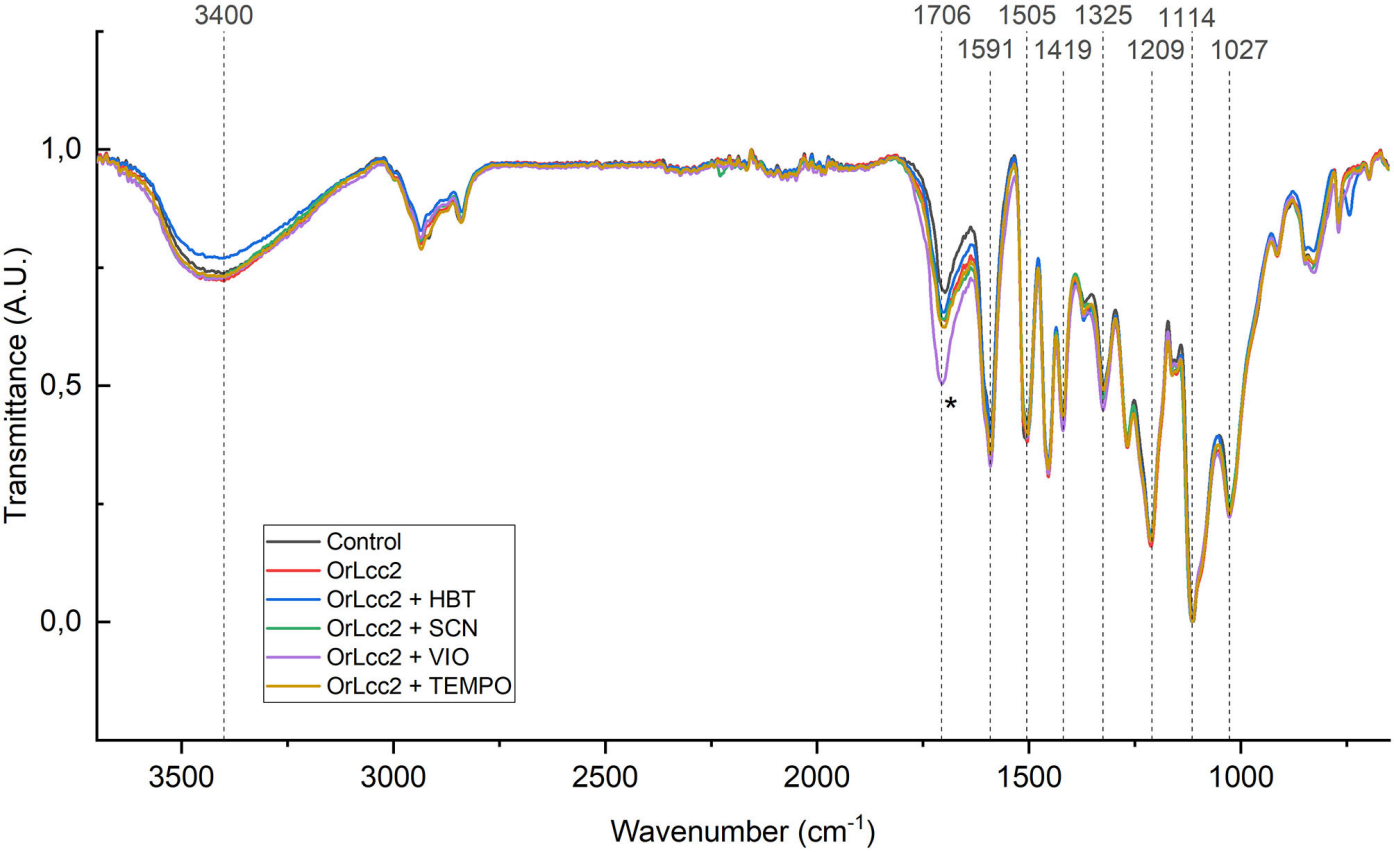
FTIR spectra of the ethanol-soluble lignin treated with *Or*Lcc2 and different mediators. The carbonyl signal area is indicated with an asterisk.

**Figure 8:**
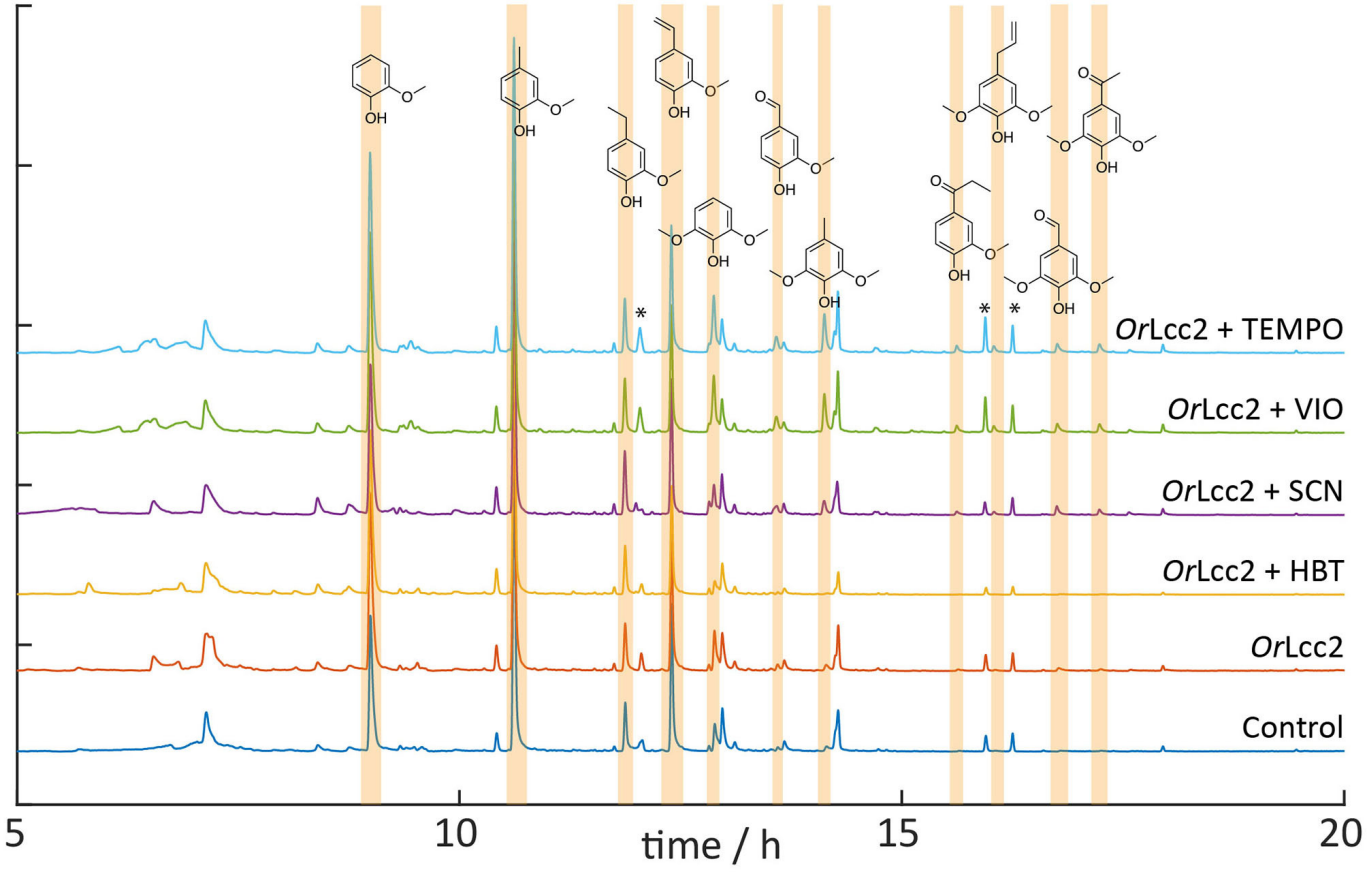
py-GC/MS chromatograms of the ethanol-soluble lignin showing the higher amounts of guaiacyl- and syringyl-oxidized fragments after treatment with *Or*Lcc2 and HBT, SCN, VIO, or TEMPO. The notable changes are highlighted in the chromatograms. Some background siloxanes and phthalates are marked with an asterisk.

**Figure 9:**
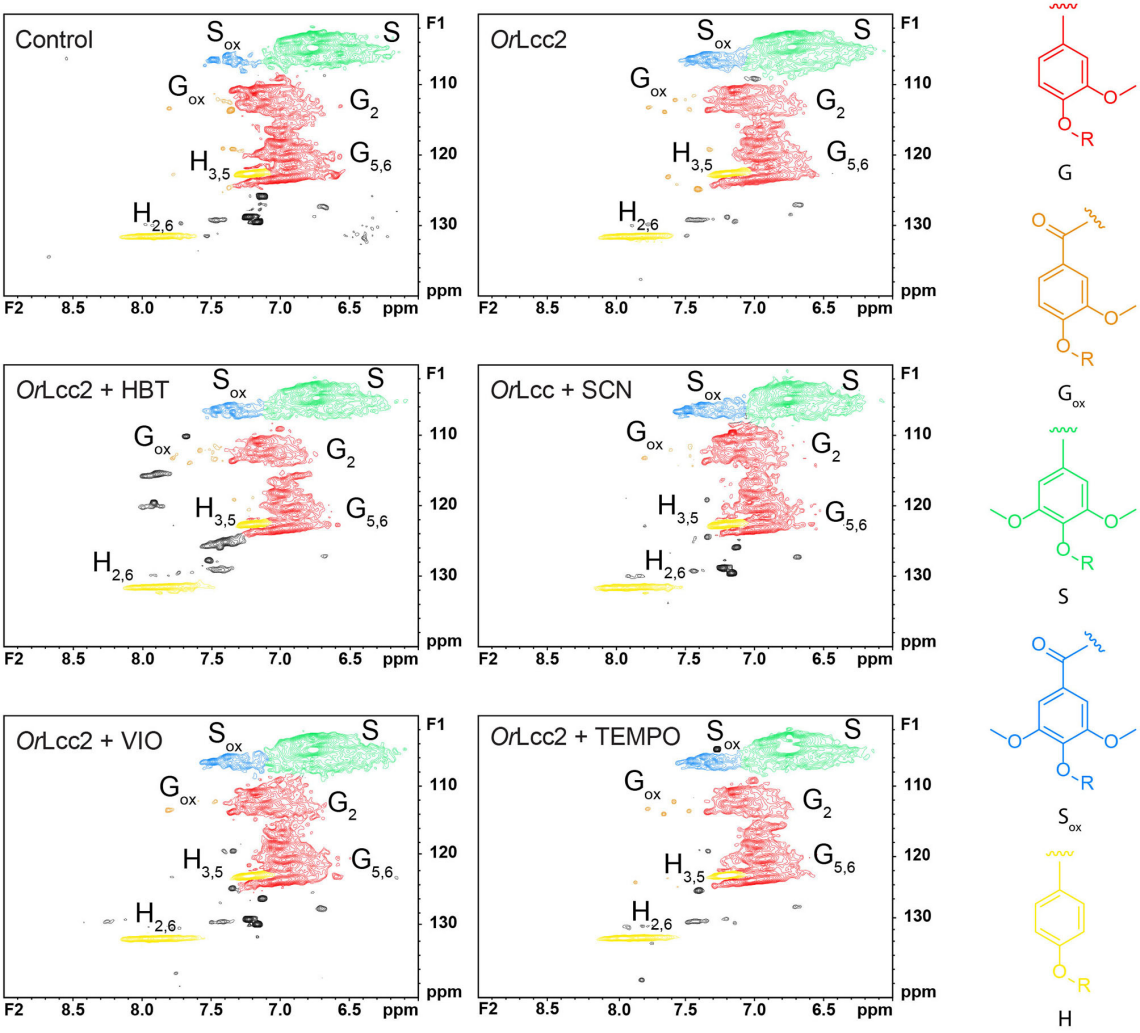
Aromatic regions of the HSQC spectra of ethanol-soluble lignin oxidized with laccase *Or*Lcc2 and different LMS. Samples were acetylated and dissolved in acetone-*d*_6_.

Because our model compound experiments showed that the LMS reactions could proceed steadily up to 120 h, the reaction times with lignins were prolonged to 72 h. The highest oxidation degrees were obtained with EL as starting substrate using mediators VIO and SCN in buffered 1,4-dioxane (20%) for 3 days. This produced a prominent amount of oxidized structures on the polymer detected by py-GC/MS, IR, and NMR analyses (Figures 7–9). However, under these conditions, changes in MWD were implying possibly more unwanted condensation reactions with ΔM_W_ 5–35% (Figure 6), and in case of reactions with increased oxygen atmosphere, the effect was even higher, up to 65% (Supplementary Figure 3). On the other hand, the material solubility can be altered also by the oxidation process itself by reducing the amount of hydroxyl groups and bringing rigidity to the molecule. This potentially results in altered solubilities of the acetylated samples in both GPC solvent THF and NMR solvent acetone-*d*_6_ and has an effect on both analysis results. The chemical aerobic TEMPO-catalyzed reactions showed a similar increase of MWD with ΔM_W_ +25%.

### FTIR Shows the Enzymatic Modification of Biorefinery Lignin

The prominent reduction of the strong absorption band present in raw material at 1,030 cm^−1^ typically originating from primary alcohol C–O bending in carbohydrates and lignin (Popescu et al., 2007) indicated that the isolated lignin fractions contained no significant amounts of hemicelluloses or cellulose (Supplementary Figure 5 and Supplementary Table 2). The EL contained a high amount of *p*-hydroxybenzoate ester groups that could be seen in the IR spectra of the control sample as typical conjugated esters at 1,707 cm^−1^ (Sammons et al., 2013), and the NMR spectral analysis confirmed the presence of these structures. The hydroxyl O–H stretching absorption peak at 3,000–3,600 cm^−1^ in control lignin with absorption maximum at 3,340 cm^−1^ was significantly higher than in the fractionated lignins EL and ABL with maximum shifted to 3,400 cm^−1^. This indicates also purification of the lignins during fractionation.

The IR spectra were measured to determine the extent of oxidation in the LMS-treated EL samples (Figure 7 and Supplementary Figure 6A). The changes in functional groups and chemical bonds present in lignin polymer can be revealed by FTIR analyses. The major changes in the IR spectra were found in the carbonyl region (1,650–1,720 cm^−1^) of the spectra. The LMS-treated samples, especially when VIO was used as a mediator, contained increased amounts of conjugated carbonyl signals, typically found in the 1,650–1,680 cm^−1^ region (Sammons et al., 2013). Even though the samples were extracted after oxidative reactions in order to remove the small-molecular-weight mediators from the reaction mixture, the presence of some residual mediators was indicated in the samples, e.g., HBT at 740 and 1,450 cm^−1^, SCN at 2,230 cm^−1^, and VIO at 1,719 cm^−1^. In particular, principal component analysis (PCA, Supplementary Figures 6B,C) revealed these deviations in the samples. The shape of the hydroxyl O–H stretching area at 3,000–3,500 cm^−1^ suggests that during the oxidation process, new carboxylic acid functionalities were not formed, suggesting that cleavage by the Bayer–Williger oxidation was not a prevalent reaction route (Figure 2B). In LMS oxidation by HBT, a decrease of hydroxyl content was observed in the reduction of the 3,000–3,500 cm^−1^ band. This may be consequence from cleavage of phenolic end-groups as illustrated in Figure 2B.

The spectra showed typical spectral absorption bands for lignin with aromatic skeletal C–H bending vibrations at 1,505 (1,512), 1,591 (1,603), and 1,419 cm^−1^ and at 1,460, 1,369, and 1,147 cm^−1^ originating from both aliphatic and aromatic C–H bending vibrations. The absorption band at 1,326 cm^−1^ can be attributed to S-groups or condensed G-groups, and bands at 1,270 and 1,210 cm^−1^ can be attributed to C–O stretching of G- and S-methoxy groups (Popescu et al., 2007; Tejado et al., 2007; Wang et al., 2019).

### Analytical py-GC/MS Reveals Oxidative Modifications of Biorefinery Lignin

Analytical pyrolysis of lignin coupled to GC/MS enables analysis of changes in the structure of LMS-treated lignins. Increases in the relative amounts of oxidized pyrolysis products, such as benzylic aldehydes and ketones, serve as an indicator of the oxidation (Ohra-aho et al., 2005). The changes in lignin structure caused by LMS oxidation were monitored by comparing the degradation profiles of the starting lignin and oxidized lignin. The pyrolytic degradation products originating from different lignin substructures were tentatively identified according to their MS spectra and comparison to the NIST spectral library and to literature (Ohra-aho et al., 2005; Brebu et al., 2013) and in some cases using reference compounds.

The pyrograms of the control lignin sample and LMS-oxidized lignins are presented in Figure 8. Lignin oxidation and degradation were detected as an increase in the amounts of oxidized phenolic pyrolysis fragments, such as vanillin, syringaldehyde, acetovanillone, and acetosyringone, that are highlighted in Figure 8. Interpretations of the signals and the changes in the relative amounts of the fragments of the *Or*Lcc2 LMS-oxidized lignins are presented in Supplementary Tables 3A,B. In general, the amounts of C2 and C3 alkyl chains (e.g., eugenol, isoeugenol, and vinylphenols) were lower in the LMS-treated samples, showing oxidative modification of the arylpropyl side chains. The amount of fragments with short-chain C1 and with no alkyl chain was generally increased after oxidative modification of the polymer backbone. The amount of guaiacol was found to increase in all samples, but 2,6-dimethoxyphenol was decreased especially with HBT, suggesting cleavage of these end-groups (Figure 2B) as also seen in the IR. As a drawback, the way the pyrolysis chamber was connected to the GC caused the high-boiling compounds to condense in the injector needle after pyrolysis, causing discrimination against the oxidized syringyl structures. This leads to underestimation of the extent of oxidation using this method. Oxidized lignin substructures tend to rearrange during the pyrolysis, hindering the analysis technique in determining also the S/G/H ratio (Jiang et al., 2017). Trace amounts of mediators were found in the chromatograms, suggesting that no extensive grafting (Munk et al., 2017) of the mediators has occurred on lignin in these experimental conditions. This was also detected as sharp signals in NMR and IR, indicating the presence of low-molecular-weight compounds.

### NMR Analysis of the Modified Biorefinery Lignins Elucidates Structural Changes

The structural changes of LMS-oxidized lignin in acid–base purified lignin were found difficult to follow using both ^13^C and 2D NMR, since the molecular weight of this lignin fraction was still quite high and the changes in the molecular backbone would have required extremely long collection times to acquire good signal-to-noise ratios. For this reason, the ethanol-extracted lignin fraction was better suited for the experiments. The samples with oxidized lignin substructures detected by py-GC/MS analyses were further analyzed by 1D (^1^H and ^13^C) and 2D NMR (HSQC and HMBC). The NMR spectra (^13^C and ^1^H–^13^C 2D correlation HSQC) of EL showed that the fraction consisted of mainly S-units together with G-units at an S/G ratio of 2:1, which is in line with the ratios of natural poplar variants (Anderson et al., 2019). The sample contained high amounts of γ-esterified *p*-hydroxybenzoate (*p*B) units typical for poplar lignin (Mansfield et al., 2012). The aliphatic oxygenated lignin side-chain region (δ_C_/δ_H_, 90–45/6.0–3.5 ppm) revealed that β-O-4 units were the major structural units together with some β-5 and β-β structures with a ratio of 6:1:1.3, respectively. The ABL showed similar S/G ratio features, and specifically, the treatments were not severe enough to cleave *p*-hydroxybenzoate esters in noticeable amounts. However, up to 6% w/w *p*-hydroxybenzoic acid could be isolated when the water-purified lignin was treated with 2 M NaOH at 130°C for 30 min. The integrations were performed from acetylated samples using their side-chain α-C-H signal areas where the signals are well-separated (Balakshin and Capanema, 2015). Practically no overlapping carbohydrate signals existed, showing that the fractionation method was efficient, but the samples contained wood extractives shown mainly at the aliphatic region (δ_C_/δ_H_, 40–15/3.5–0.5 ppm).

The benzylic oxidation of the phenylpropanoic side chain is more conveniently studied by following changes in HSQC aromatic correlation region (δ_C_/δ_H_, 100–160/8.5–6.0 ppm) shown in Figure 9. The most convenient detection of oxidized benzylic positions is obtained by following the changes in chemical shifts of S-units S2/6 (δ_C_/δ_H_ 104.0/6.7 ppm) in the aromatic area of HSQC spectra where oxidized S2/6_ox_ signals shifted to δ_C_/δ_H_ 106.0/7.4 ppm (Figure 9). The ratio of these signals clearly increased during the laccase and LMS treatment in comparison to the control sample. The corresponding G2/6 signals at δ_C_/δ_H_ 110.8/7.0 ppm also showed a similar shift G2_ox_ at δ_C_/δ_H_ 110/7.5 and G6_ox_ 124/7.6 ppm, but to a lesser extent (Kim and Ralph, 2010; Ralph and Landucci, 2010; JiaLong et al., 2013; Rahimi et al., 2013). The carbonyl correlation peaks in the HMBC spectra confirmed that the oxidized region is correctly delineated. To have some estimation on the degree of oxidation, the contour volume integrals were measured for S2/6 and the corresponding oxidized signals showing 9–12% oxidation of S-units and minimal oxidation of G-units (data not shown). The quantification of the signals was approximations because the polymer end-groups give higher integration values due to their much slower relaxation compared to the rest of the lignin polymer (Mansfield et al., 2012). The amount of oxidation expectedly increased already with laccase treatment without addition of mediators, because the one-electron oxidation of lignin phenolic end-groups produces radicals that can transfer the oxidation further to the rest of the polymer serving as a mediator. However, the values support results obtained with py-GC/MS (Supplementary Tables 3A,B) and IR analysis (Figure 7 and Supplementary Table 2), showing that SCN and VIO produced the highest yield of carbonyl structures but also that HBT and TEMPO showed increased oxidation levels. The ^1^H NMR integrations gave a crude estimation with considerably higher 15–35% overall (S- and G-units) oxidation values (Supplementary Table 4).

In EL, some aldehyde carbonyl correlation signals could be detected at δ_C_/δ_H_ 194/9.5 ppm as shown in the HSQC and long-range HMBC correlation signals, suggesting that EL contained low-molecular-weight aldehydes either originating from wood extracts or formed during steam-explosion treatment. The aromatic region correlations to benzylic ketone carbonyls increased with LMS, and the highest amount of correlation signals was present in the LMS samples treated with mediators SCN and VIO. Particularly, in the case of the benzylic ketones at 196 ppm, a correlation signal to the aliphatic side-chain region in the F1 dimension was detected (Ralph and Landucci, 2010).

The biocatalytic reactions were compared to chemical reaction catalyzed by FeIII(NO_3_)_3_-TEMPO in 1,4-dioxane or formic acid. The resulting NMR spectra of the isolated products showed similar signs of oxidation as the LMS-oxidized products (Supplementary Figures 7, 8). In 1,4-dioxane, around 27% oxidation of S-units was evaluated with HSQC contour integration, and interestingly, the amount of oxidized G-units seemed to be higher (32%) when using chemical TEMPO catalysis. In formic acid, the oxidation process yielded a solid fraction (60%) with low oxidation levels and an ethyl acetate extractable fraction (30%) with a high amount of oxidized structures, but isolated solid fraction contained 17% oxidized S-units and 14% G-units. The soluble fraction contained 41% oxidized S- and 18% G-units.

Interestingly, both N–OH-type and syringyl-type phenol mediators were found effective for oxidation of lignin, but more research on mediator preference and their optimum performances is needed. Also, the purified lignin fractions contained high amounts of side-chain *p*-hydroxybenzoic acid esters, increasing the steric hindrance in lignin structure for mediated benzylic oxidation, which could be reduced by hydrolysis of the starting material. The comparison with chemical catalysis (using an ionic mechanism) showed that the mediated oxidation (using mainly HAT) prefers oxidation of benzylic S-type aromatics over G-type aromatics, suggesting that in HAT, especially, stabilization of the formed carbon radicals is important.

The side-chain signals in oxidized lignin were found challenging to detect in our experiments, as similarly reported in literature (Rahimi et al., 2013; Longe et al., 2018; Rinesch and Bolm, 2018; Song et al., 2019). More accurate determination of oxidation degree would have required higher sample amounts and quantitative pulse sequences with longer acquisition times. Though oxidation of material was clearly shown at the aromatic signal area, confirming the results obtained by py-GC/MS and IR analyses, higher oxidation levels could be obtained by optimizing the reaction systems [e.g., by immobilizing the laccases to increase their stability and resistance (Ba et al., 2013; Gasser et al., 2017)] or by genetically modifying their properties to better tolerate reaction conditions with elevated temperatures, high organic solvent concentrations, and extreme pH (Mate and Alcalde, 2015).

## Conclusions

In this study, the applicability of two laccases from *O. rivulosa* was assessed for biotechnical applications in cellulosic biorefinery and pulp-and-paper sector at low-pH reaction conditions in the presence of organic cosolvents. Lignin model compounds were used to study the preference of the enzymes toward different mediators as well as the tolerance of the system toward organic solvents in a low-pH environment.

The LMS with *O. rivulosa* laccases was studied on isolated biorefinery poplar lignin fractions in order to assess the applicability of LMS in lignin modification reactions for further lignin valorization. The laccases were found to oxidize lignin fractions, and the desired selective benzylic α-oxidation was observed in optimized reaction conditions. Specifically, VIO was found to be stable and effective in obtaining modified lignin fractions that could be used for further modifications in biorefinery. Surprisingly, also the phenolic mediator SCN was found to be almost as effective as VIO, based on the analyses by IR, py-GC/MS, and NMR. When the biocatalytic reactions were compared to catalytic TEMPO systems with Fe(III)nitrate, the biocatalyzed reactions were found to predominantly oxidize lignin S-units, whereas chemically catalyzed reactions oxidized both S- and G-units.

## Data Availability Statement

The raw data supporting the conclusions of this article will be made available by the authors, without undue reservation.

## Author Contributions

JK, PN, and JS planned the experiments. JK carried out the experiments and took the lead in writing the manuscript. RM and JM contributed to sample analysis. JK, PN, RM, JM, and JS contributed to the interpretation of the results. KH and MK produced the enzymes. PN, JS, MM, and KH provided feedback and helped shape the research and manuscript. All authors contributed to the article and approved the submitted version.

## Conflict of Interest

The authors declare that the research was conducted in the absence of any commercial or financial relationships that could be construed as a potential conflict of interest.
