# Supplementary material for: Applicability of Recombinant Laccases From the White-Rot Fungus Obba rivulosa for Mediator-Promoted Oxidation of Biorefinery Lignin at Low pH
Source: Front Bioeng Biotechnol. 2020 Dec 17;8:604497. doi: 10.3389/fbioe.2020.604497 (PMC7773891; doi:10.3389/fbioe.2020.604497)
Supplement: Supplementary file 1 [file Data_Sheet_1.docx]

Supplementary Material

**Supplementary Table 1**. Lignin oxidation experiments by recombinant laccases from *O. rivulosa.* Effect on molecular weight distribution is expressed as the difference ∆M_N_ or ∆M_W_ between non-treated lignin fraction (ABL or EL) and laccase (Lcc) or laccase-mediator system (LMS) treated samples.

| Laccase | Lignin purification method | pH | Co-solvent | Mediators | Analysis | Solubility and reaction time | Effect on MW distribution |
| --- | --- | --- | --- | --- | --- | --- | --- |
| *Obba rivulosa* Lcc2 | ABL | 3 | 20% 1,4-dioxane | HBT, MeS | GPC, IR, Py-GCMS | Low; 24 h | Lcc ∆M_N_ 10%; ∆M_W_10%  LMS ∆M_N_ 1- -8%; ∆M_W_2-10% |
| *Obba rivulosa* Lcc2 | ABL | 3 + 4 | 50% EtOH | HBT, MeS, | GPC, IR, Py-GCMS | Low; 24 h | Lcc ∆M_N_ -25%; ∆M_W_-16%  LMS ∆M_N_ 1- -19%; ∆M_W_16 - -11% |
| *Obba rivulosa* Lcc2 | ABL | 3 | 50% EtOH | HBT, MeS, HPI, VIO, TEMPO and ABTS | GPC, IR, Py-GCMS, NMR | Low; 3 d | Lcc ∆M_N_ 0%; ∆M_W_-3%  LMS ∆M_N_ 5- -20%; ∆M_W_10 - -10% |
| *Obba rivulosa* Lcc2 | EL | 3,5 | 20% 1,4-dioxane | HBT, SCN, VIO, TEMPO | GPC, IR, Py-GCMS, NMR | Moderate; 3 d | Lcc ∆M_N_ +10%; ∆M_W_+15%  ∆M_N_6- 18%: ∆M_W_5- 35% |
| *Obba rivulosa* Lcc1 | EL | 3 | 20% 1,4-dioxane | HBT, SCN, VIO | GPC, (IR), Py-GCMS | Moderate; 3 d | Lcc ∆M_N_ +3%; ∆M_W_+5%  LMS 7 - -8%; ∆M_W_11- -9% |
| *Obba rivulosa* Lcc2  + O_2_ | EL | 3,5 | 20% 1,4-dioxane | VIO, SCN | GPC, IR, Py-GCMS | Moderate; 1 d | Lcc∆M_N_30%; ∆M_W_55%  LMS ∆M_N_15-20%; ∆M_W_30-65% |


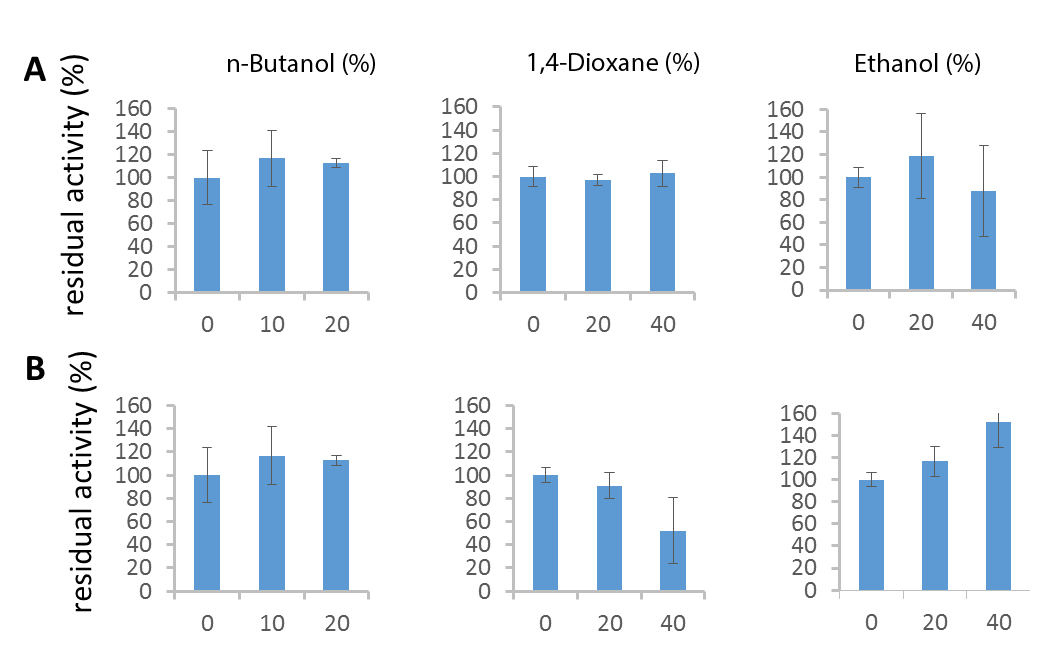


**Supplementary Figure 1**. Solvent tolerance of OrLcc1 (A) and OrLcc2 (B). The residual activity after 2.5 h incubation was measured by using ABTS as the substrate at pH 3.0. The measurements were carried out as duplicates and averages are plotted. Residual activities are calculated as percentage of the initial laccase activities assayed in aqueous buffer without organic solvents.

**Supplementary Figure 2**. Oxidation of internal standard 1,3,5-trimethoxybenzene TMB in the presence of ABTS (*Or*Lcc2)-laccase-mediator system showing that TMB was readily consumed up to 80% in the reaction mixture within 6 h.


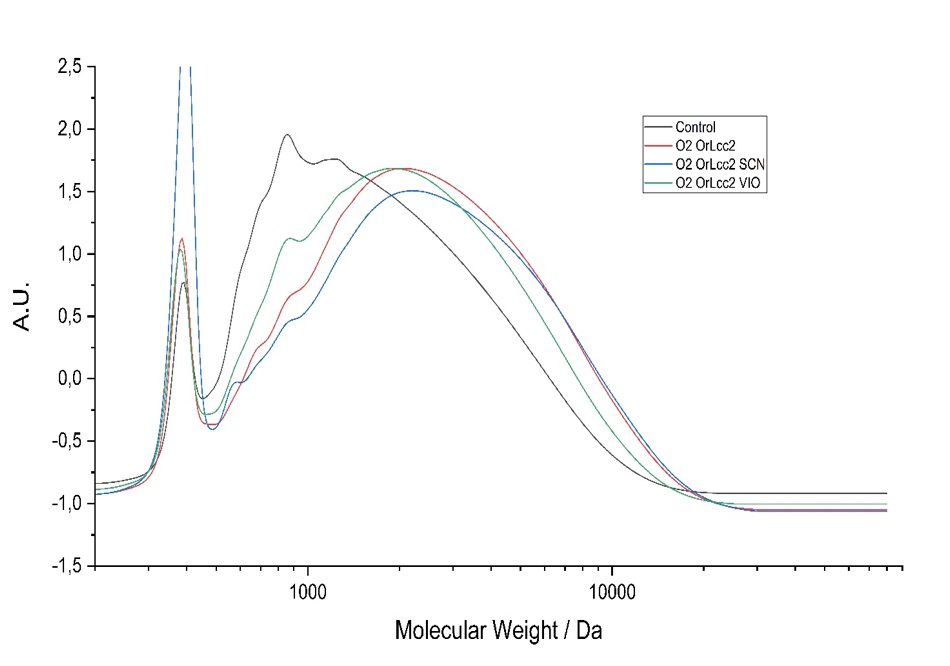


| Sample | M_N_ | M_W_ | ΔM_N_ | ΔM_W_ | ΔM_N_-% | ΔM_W_-% |
| --- | --- | --- | --- | --- | --- | --- |
| Control | 1140 | 1920 | 0 | 0 | 0 | 0 |
| *Or*Lcc2 | 1460 | 2970 | 320 | 1040 | 30 | 55 |
| *Or*Lcc2 + SCN | 1390 | 3210 | 240 | 1280 | 20 | 65 |
| *Or*Lcc2 + VIO | 1320 | 2490 | 170 | 570 | 15 | 30 |

**Supplementary Figure 3**. Effect of LMS on the molecular weight distribution of EL lignin oxidized by *Or*Lcc2 at pH 3.5 with 20% dioxane as co-solvent and under oxygen atmosphere.





*

*

*

Aromatic region

Aliphatic side chain region

Aliphatic extractives region

**Supplementary Figure 4**.^1^H NMR spectra from the ethyl acetate soluble fractions of different LMS treatments of the ethanol soluble lignin. The spectra show low amounts of lignin fragments, extractives and mediators that are highlighted with an asterisk. VIO and TEMPO are not detectable in the ^1^H NMR spectra. The non-acetylated samples were analyzed in acetone-d_6_.


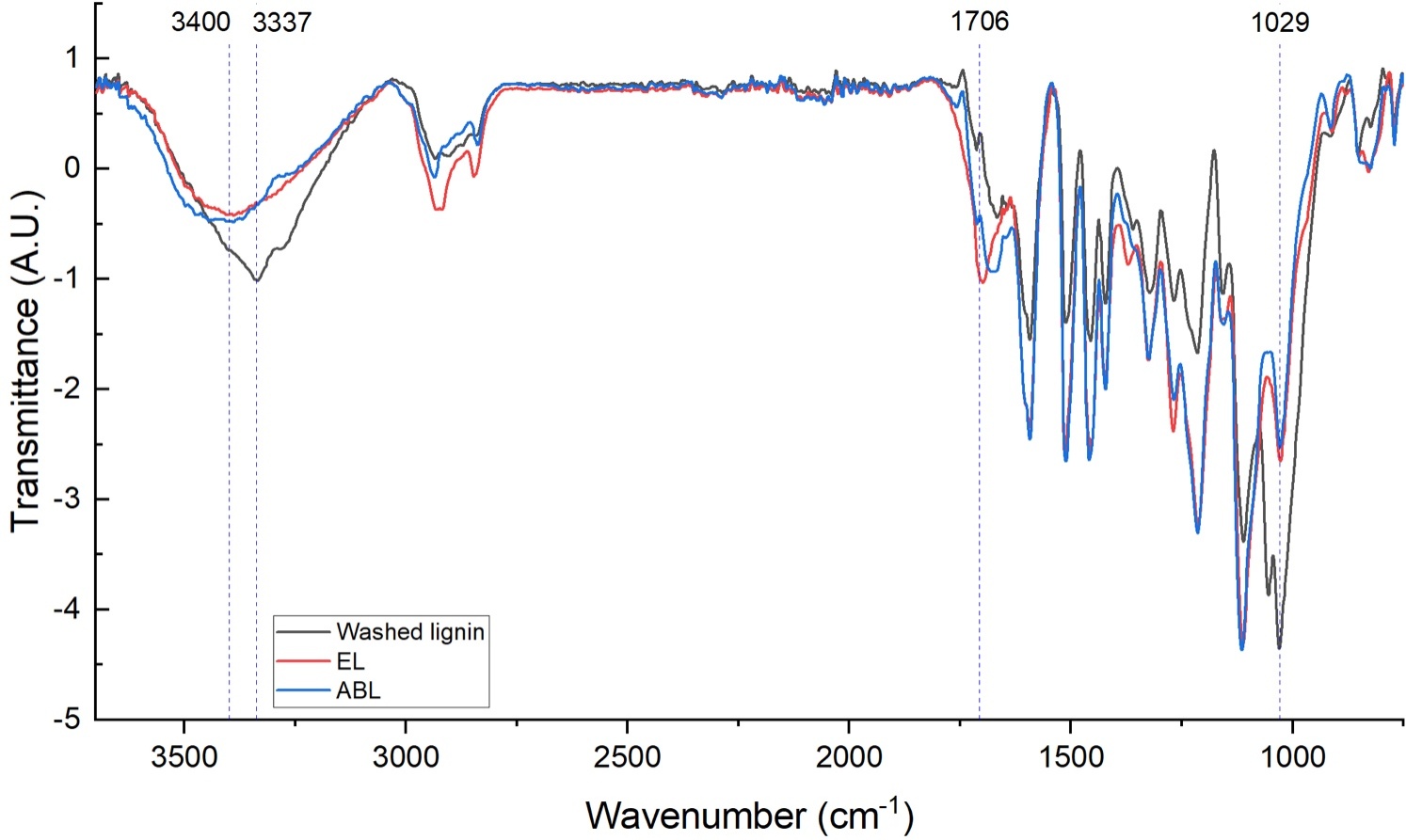


**Supplementary Figure 5**. The IR spectra of washed lignin, ethanol soluble and acid-base-fractionated lignin. The main differences can be found in hydroxyl O-H stretching region at 3000-3600 cm^-1^ and aliphatic O-H and ether C-O stretching region at 1028 cm^-1^indicating reduction in the carbohydrate content after fractionation. Especially EL contained high amount of *p*-hydroxybenzoate ester groups that could be seen in the IR-spectra of control sample as typical conjugated esters at 1707 cm^-1^.

**Supplementary Table 2** Analysis of lignin functional groups detected by FT-IR

| Functional group | | In spectra  Wave number/cm^−1^ |
| --- | --- | --- |
| Hydroxyl O-H stretching | 3000-3600 | |
| Aromatic C-H stretching | >3000 | |
| Aliphatic C-H stretching | 2900-3000 | |
| Non-conjugated C=O stretching | 1715 | |
| Conjugated esterC=O stretching | 1707 | |
| Conjugated carbonyl C=O stretching | 1650-1690 | |
| Aromatic C=C stretching | 1603, 1591 | |
| Aromatic C=C stretching | 1512, 1505 | |
| C-H deformations in phenol rings | 1418 | |
| C-O vibrations | 1326 | |
| Guaiacyl ring breathing, C–O in aromatic methoxyl groups | 1270 | |
| C-O stretching of G- and S-methoxy groups | 1210 | |
| Aromatic C–H in-plane deformation (typical for S units), C=O stretch | 1112 | |
| Aliphatic O-H and ether C-O stretching | 1028 | |
| Aromatic C–H out-of-plane deformations | 834 | |


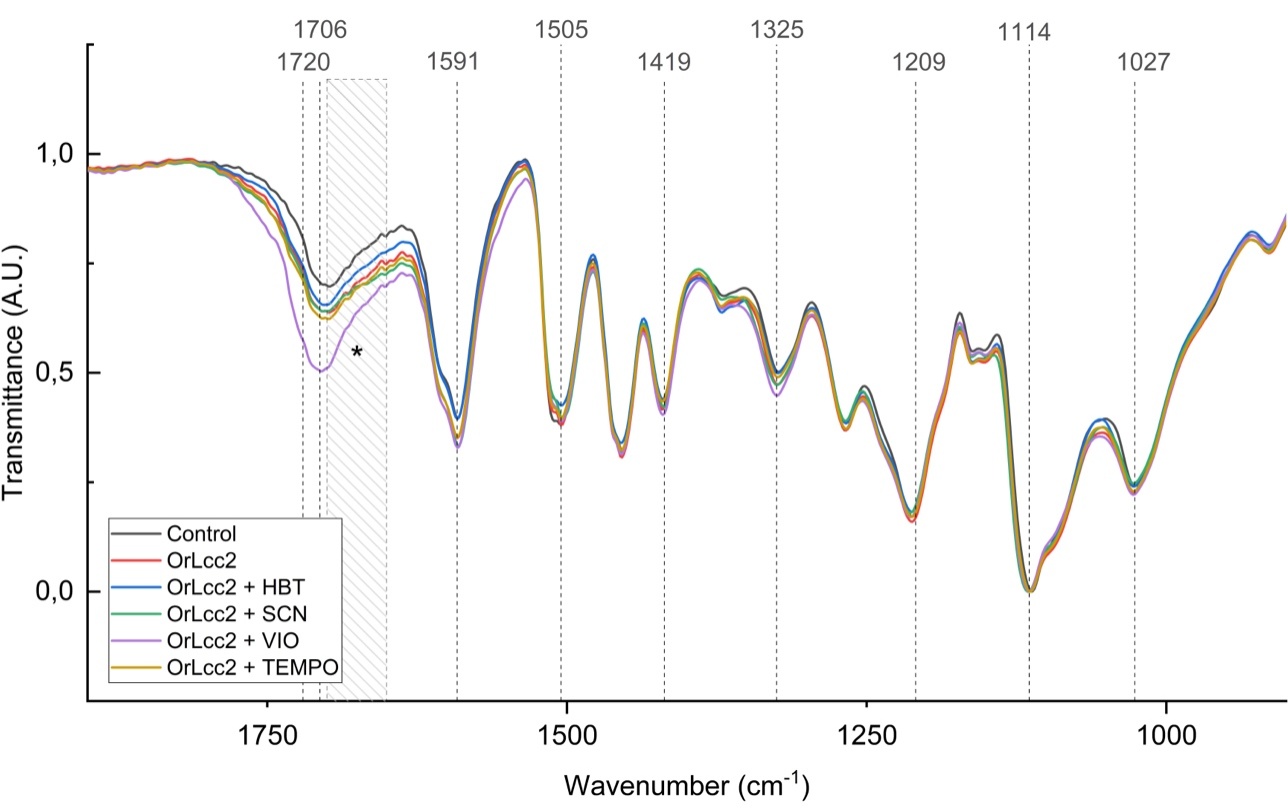


**Supplementary Figure 6A**. Infrared spectral analysis at 1800 – 750 cm-^1^ showing only minor differences in the IR ‘fingerprint region’. The highest differences can be found in the carbonyl region compared to non-treated ethanol soluble lignin spectra (black line). The amount of carbonyl functionalities in LMS-treated samples has been increased, but due to polymeric nature of lignin, the changes in the molecular backbone can be seen as widening of the peaks. Non-conjugated carbonyls can be found > 1700 cm^-1^ and conjugated carbonyls representing new oxidized structures formed during LMS-treatments between 1650-1700 cm^-1^.





**Supplementary Figure 6B.** PCA scores plot of the IR-analysis shows that oxidation of lignin by HBT produced material that differs from other LMS-treated samples. The laccase-oxidized sample resembled most closely the non-treated control sample.





**Supplementary Figure 6C.** PCA loadings spectra and IR-spectrum of the control material. The presence of some residual mediators was indicated in the samples, e.g. HBT at 740 and 1450 cm^-1^, and VIO at 1719 cm^-1^.

**Supplementary Table 3A**. Pyrolysis-GC/MS analysis tables shows the most important fragments and their relative amounts. C indicates the chain length of aromatic alkyl phenol fragments. RT shows the retention time of each fragment.

| **RT** | **Name** | **Control** | **Laccase** | **HBT** | **SCN** | **VIO** | **TEMPO** | **C** |
| --- | --- | --- | --- | --- | --- | --- | --- | --- |
| 10,28 | **1,3-Dimethoxybenzene** | 0,005 | 0,025 | 0,067 | 0,157 | 0,086 | 0,037 | 1 |
| 15,21 | **1-(4-hydroxy-3-methoxyphenyl)-2-propanone** | 0,000 | 0,000 | 0,000 | 0,074 | 0,077 | 0,000 | 3 |
| 9,32 | **2,3-Dimethylanisole** | 0,167 | 0,581 | 0,288 | 0,550 | 0,329 | 0,175 | 1 |
| 9,95 | **2,4-Dimethylphenol** | 0,125 | 0,075 | 0,263 | 0,885 | 0,084 | 0,084 | 1 |
| 13,26 | **2,5-Dimethoxybenzyl alcohol** | 0,050 | 0,070 | 0,028 | 0,040 | 0,124 | 0,033 | 1 |
| 14,12 | **2,6-Dimethoxy-4-methylphenol** | 0,396 | 0,755 | 0,042 | 1,889 | 4,193 | 1,393 | 1 |
| 11,53 | **2,6-Dimethoxytoluene** | 0,013 | 0,108 | 0,064 | 0,167 | 0,031 | 0,135 | 1 |
| 12,87 | **2,6-dimethoxyphenol** | 3,756 | 4,867 | 2,057 | 2,974 | 6,192 | 5,481 | 1 |
| 9,88 | **2-Methoxy-3-methylphenol** | 1,997 | 2,120 | 0,000 | 0,016 | 0,000 | 0,000 | 1 |
| 12,40 | **2-Methoxy-4-vinylphenol** | 16,048 | 14,122 | 12,357 | 12,666 | 10,692 | 13,396 | 2 |
| 8,39 | **2-methylphenol** | 0,455 | 1,123 | 1,340 | 2,802 | 1,462 | 0,901 | 1 |
| 11,28 | **3,4-Dimethoxytoluene** | 0,000 | 0,000 | 0,000 | 0,071 | 0,000 | 0,000 | 1 |
| 11,74 | **3,5-Dimethoxytoluene** | 0,374 | 0,484 | 0,618 | 0,792 | 0,583 | 0,373 | 1 |
| 12,82 | **3-Methoxy-2,5,6-trimethylphenol** | 0,387 | 0,420 | 0,473 | 0,888 | 0,347 | 0,315 | 1 |
| 11,63 | **4-Ethyl-2-methoxyphenol** | 5,785 | 5,366 | 5,832 | 0,051 | 4,984 | 5,394 | 2 |
| 15,11 | **4-Ethylsyrol** | 0,000 | 0,000 | 0,000 | 0,054 | 0,054 | 0,000 | 2 |
| 8,77 | **4-Methylphenol** | 0,603 | 0,635 | 0,509 | 1,406 | 1,003 | 1,001 | 1 |
| 15,62 | **4-Vinylsyringol** | 0,053 | 0,026 | 0,000 | 0,373 | 0,629 | 0,056 | 2 |
| 17,57 | **Acetosyringone** | 0,000 | 0,019 | 0,000 | 0,319 | 0,259 | 0,050 | 2 |
| 14,69 | **Acetovanillone** | 0,000 | 0,128 | 0,000 | 0,170 | 0,377 | 0,105 | 2 |
| 10,62 | **Creosol** | 37,655 | 35,009 | 38,539 | 35,903 | 31,334 | 31,012 | 1 |
| 12,96 | **Eugenol** | 4,010 | 3,039 | 2,921 | 3,168 | 2,054 | 2,838 | 3 |
| 8,99 | **Guaiacol** | 20,637 | 25,902 | 26,554 | 23,691 | 25,018 | 30,379 | 1 |
| 16,04 | **Methoxyeugenol** | 0,000 | 0,000 | 0,000 | 0,249 | 0,537 | 0,000 | 3 |
| 7,14 | **Phenol** | 6,008 | 3,263 | 6,836 | 6,227 | 5,416 | 5,541 | 1 |
| 16,75 | **Syringaldehyde** | 0,000 | 0,151 | 0,005 | 1,111 | 0,945 | 0,301 | 1 |
| 13,57 | **Vanillin** | 0,000 | 0,330 | 0,000 | 1,184 | 1,571 | 0,000 | 1 |
| 16,63 | **cis-Methoxyisoeugenol** | 0,000 | 0,000 | 0,000 | 0,007 | 0,000 | 0,000 | 3 |
| 13,66 | **cis-isoeugenol** | 0,965 | 0,782 | 0,630 | 0,742 | 0,489 | 0,678 | 3 |
| 13,10 | **p-Propylguaiacol** | 0,512 | 0,511 | 0,571 | 0,781 | 0,291 | 0,246 | 3 |
| 17,23 | **trans-Methoxyisoeugenol** | 0,000 | 0,089 | 0,005 | 0,593 | 0,840 | 0,074 | 3 |

**Supplementary Table 3B**. Py-GC/MS table with differences calculated on control - LMS-oxidized lignins. The highest increase of the peaks is indicated with green and highest decrease is indicated with red. In general, the amount of C2 and C3 alkyl chains (e.g. eugenol, isoeugenol, vinylphenols) were lower in the LMS-treated samples showing oxidative modification of the arylpropyl side-chains. The amount of fragments with short chain C1 and with no alkyl chain, was generally increased after oxidative modification of the polymer backbone. The amount of guaiacol was found to increase in all samples, but 2,6-dimethoxyphenol was decreased especially with HBT suggesting cleavage of these end-groups.

**Supplementary Table 4**. ^1^H NMR-integration results compared with oxidized aromatic region 7.7-7.2 to regular aromatic region 7.2-6.3.

| *Or*Lcc2 |  |  |  |  |  |
| --- | --- | --- | --- | --- | --- |
|  |  | Oxidized7.7-7.2 | Regular7.2-6.3 | Change | notes |
| 1 | Ctrl | 0,236 | 1 | 100 % |  |
| 2 | Lcc | 0,164 | 1 | 70 % |  |
| 3 | HBT | 0,299 | 1 | 130 % | Contains HBT; overestimates change |
| 4 | SCN | 0,313 | 1 | 135 % | Contains SCN; underestimates change |
| 5 | VIO | 0,268 | 1 | 115 % |  |
| 6 | TEMPO | 0,171 | 1 | 70 % |  |


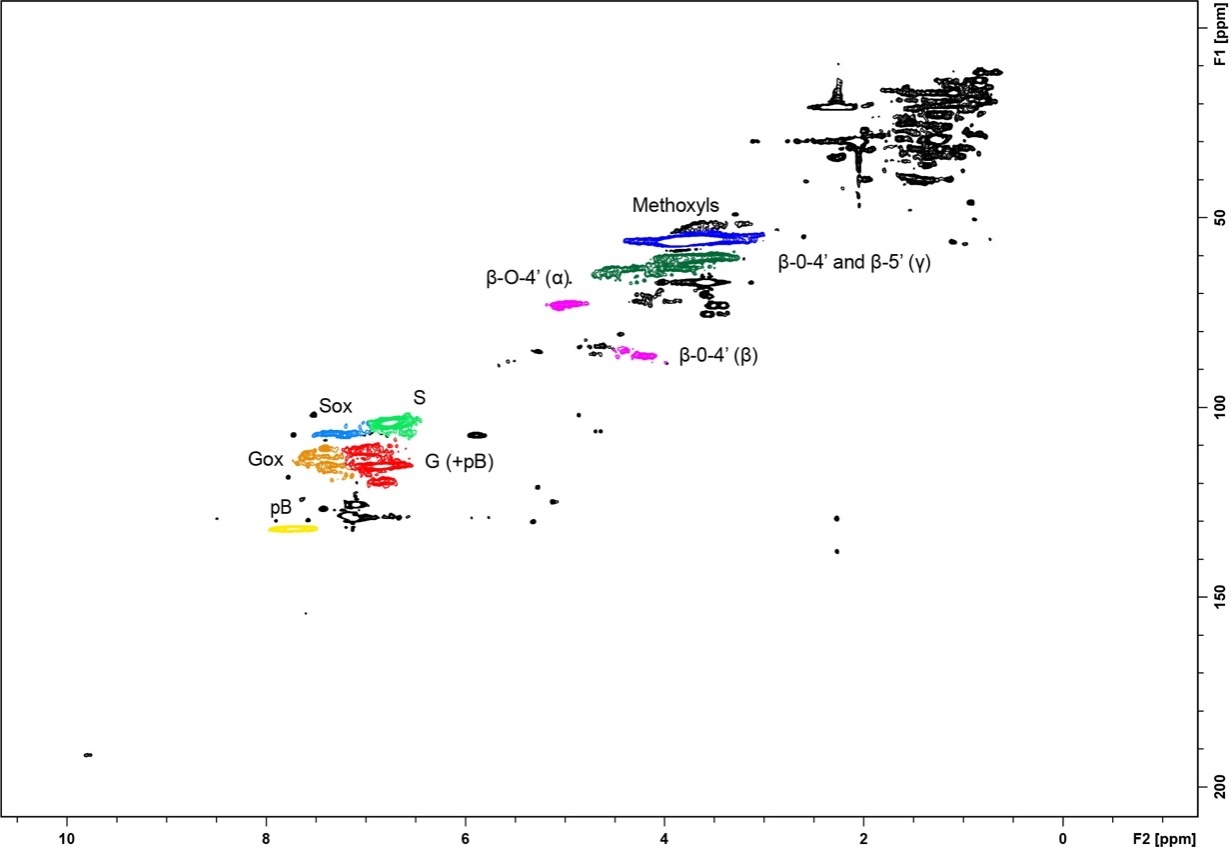


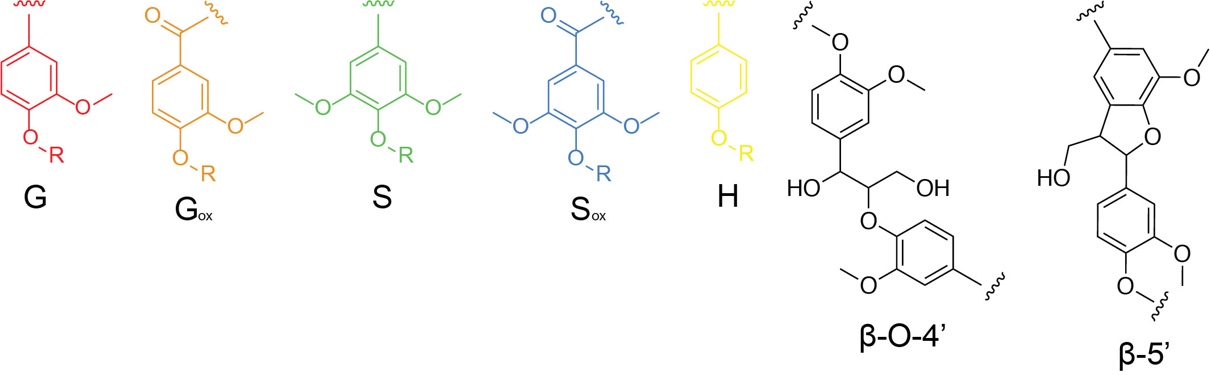


**Supplementary Figure 7**. The metal-catalyzed aerobic Fe(III)NO_3_ -TEMPO oxidation of ethanol solublelignin in 1,4-dioxane. The non-acetylated sample was analyzed in acetone-d_6_.

A)


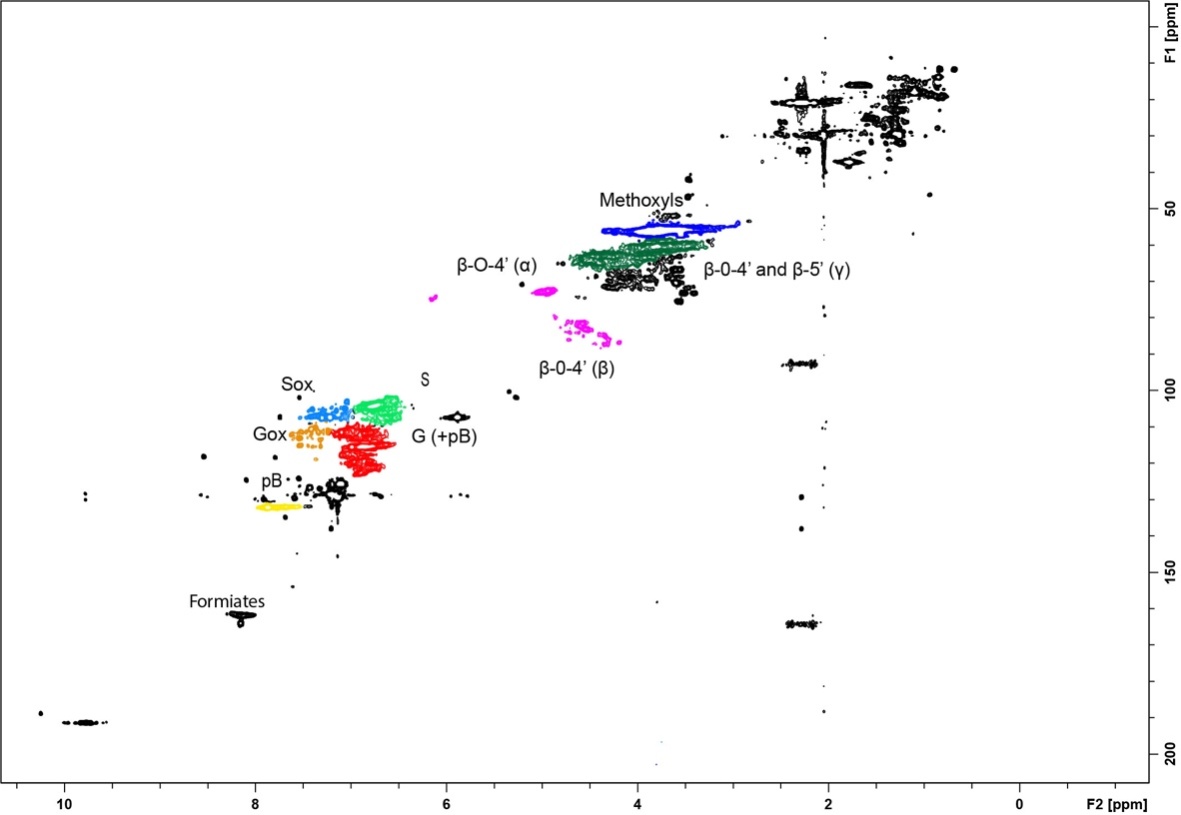


B)


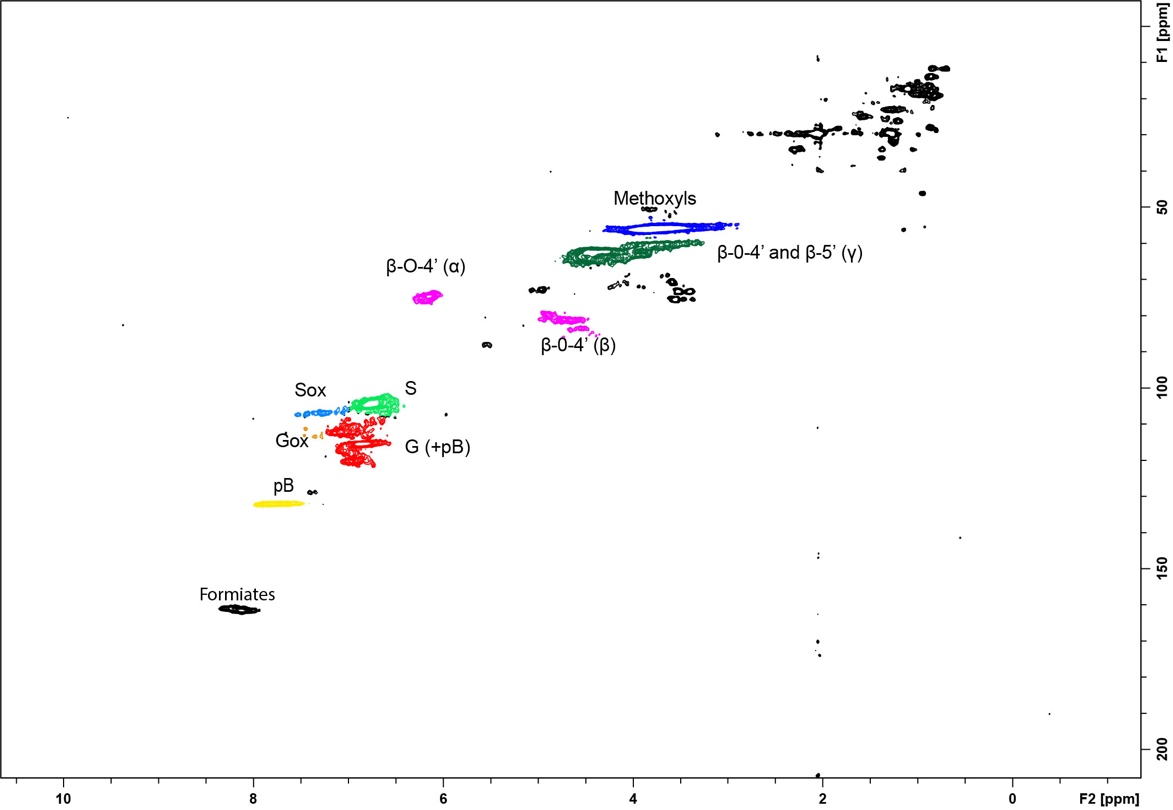


**Supplementary Figure 8**. The metal-catalyzed aerobic Fe(III)NO_3_ -TEMPO oxidation of ethanol solublelignin in formic acid A) fraction extracted in ethyl acetate B) fraction isolated by precipitation. The samples were analyzed in acetone-d_6_. The spectra indicate that the propyl side chain hydroxyls were formylated during the treatment.
